# Viral vector and nucleic acid vaccines against COVID-19: A narrative review

**DOI:** 10.3389/fmicb.2022.984536

**Published:** 2022-08-31

**Authors:** Saeed Khoshnood, Roya Ghanavati, Maryam Shirani, Hossein Ghahramanpour, Mohammad Sholeh, Aref Shariati, Nourkhoda Sadeghifard, Mohsen Heidary

**Affiliations:** ^1^Clinical Microbiology Research Center, Ilam University of Medical Sciences, Ilam, Iran; ^2^Student Research Committee, Ilam University of Medical Sciences, Ilam, Iran; ^3^School of Paramedical Sciences, Behbahan Faculty of Medical Sciences, Behbahan, Iran; ^4^Toxicology Research Center, Medical Basic Sciences Research Institute, Ahvaz Jundishapur University of Medical Sciences, Ahvaz, Iran; ^5^Department of Bacteriology, Faculty of Medical Sciences, Tarbiat Modares University, Tehran, Iran; ^6^Department of Microbiology, Pasteur Institute of Iran, Tehran, Iran; ^7^Molecular and Medicine Research Center, Khomein University of Medical Sciences, Khomein, Iran; ^8^Department of Laboratory Sciences, School of Paramedical Sciences, Sabzevar University of Medical Sciences, Sabzevar, Iran; ^9^Cellular and Molecular Research Center, Sabzevar University of Medical Sciences, Sabzevar, Iran

**Keywords:** SARS-CoV-2, COVID-19, DNA vaccine, RNA vaccine, review

## Abstract

After about 2 years since the first detection of severe acute respiratory syndrome coronavirus 2 (SARS-CoV-2) infections in Wuhan, China, in December 2019 that resulted in a worldwide pandemic, 6.2 million deaths have been recorded. As a result, there is an urgent need for the development of a safe and effective vaccine for coronavirus disease 2019 (COVID-19). Endeavors for the production of effective vaccines inexhaustibly are continuing. At present according to the World Health Organization (WHO) COVID-19 vaccine tracker and landscape, 153 vaccine candidates are developing in the clinical phase all over the world. Some new and exciting platforms are nucleic acid-based vaccines such as Pfizer Biontech and Moderna vaccines consisting of a messenger RNA (mRNA) encoding a viral spike protein in host cells. Another novel vaccine platform is viral vector vaccine candidates that could be replicating or nonreplicating. These types of vaccines that have a harmless viral vector like adenovirus contain a genome encoding the spike protein of SARS-CoV-2, which induces significant immune responses. This technology of vaccine manufacturing has previously been used in many human clinical trials conducted for adenoviral vector-based vaccines against different infectious agents, including Ebola virus, Zika virus, HIV, and malaria. In this paper, we have a review of nucleic acid-based vaccines that are passing their phase 3 and 4 clinical trials and discuss their efficiency and adverse effects.

## Introduction

**Figure 1:**
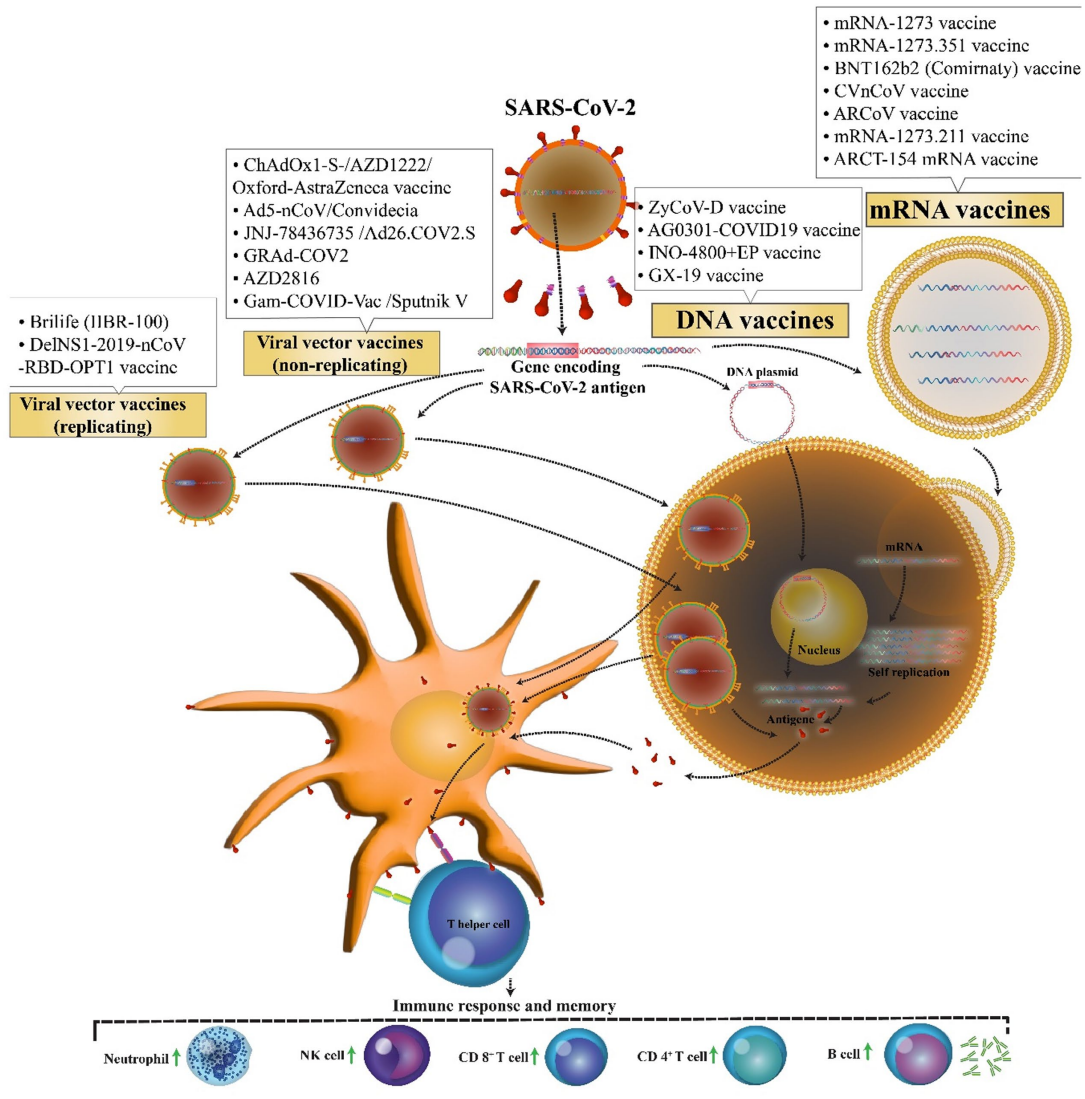
Nucleic acid-based vaccine candidates against COVID-19.

The first novel coronavirus disease 2019 (COVID-19) epidemic broke out in Wuhan and became rapidly widespread with confirmed cases in whole China and the world and has caused a new global public health crisis (Zhu et al., 2020). However, from December 2019 to July 2022, it has led to more than 553 million coronavirus cases and more than 6.3 million deaths worldwide (WHO, 2022). Dramatic increase in the prevalence of the virus and also the growth in mortality rate resulted in a global competition for the discovery of the virus vaccine among researchers. Perhaps, finding a potential vaccine for the prevention of COVID-19 is a large step forward toward ending this deadly pandemic. Therefore, more than 71 vaccine candidates are in phase 3 clinical trials, and only 38 vaccines with different platforms have been approved. Nucleic acid vaccines benefit genetic instructions in the form of DNA or messenger RNA (mRNA), to encode specific pathogenic antigens after entering the host cells like dendritic cells and macrophages. These cells present viral proteins to CD4 + T-helper cells through the major histocompatibility complex (MHC) class 1, resulting in the secretion of IL-12 and IFN-γ to further Th1 and natural killer cells activation (Figure 1; Chavda et al., 2021). Most of these vaccines contained a plasmid encoding the spike (S) glycoprotein genes. The advantages of these vaccines are the ability to stimulate simultaneous innate and adaptive immune responses, safety, scalability for mass production, and cost-effectiveness, and ease of manufacturing (Abadi et al., 2018). Easy manipulation of vaccine nucleic acid has superiority over other vaccines against various likely mutations of SARS virus (Silveira et al., 2021).

Research has demonstrated that before the emergence of the COVID-19 pandemic, no existing licensed vaccine has used this technology. DNA vaccines insert a piece of specific genes encoding an antigen plus their promoter into the host cell, using DNA plasmids as a vector. For delivery to cells, the plasmid DNA needs to cross *via* the nuclear pores of the host cell, a significant barrier for DNA-based therapeutics. Electroporation (EP) and/or gene guns are technologies that not only can facilitate the delivery of plasmid DNA but also can greatly elevate the expression of the target protein (Khavari et al., 2016). In addition, one of the benefits of DNA vaccines rather than mRNA vaccines is greater thermal stability and less refrigeration requirements (Kim and Jacob, 2009). Nevertheless, DNA vaccines have to integrate into the host genome, thereby increasing the risk of insertional mutations (Liu, 2019). The mRNA vaccine encodes the antigen genome of interest in an mRNA or self-amplifying mRNA, which can be translated into the cytoplasm of the host cells after vaccination. The vaccine also does not need to integrate into the host genome like the DNA one; therefore, it cannot change or influence the host genome, as one of the potential practical advantages of mRNA vaccines (Park et al., 2021). Moreover, utilizing *in vitro* transcription does not need to be multiplied in bacteria, or cell culture makes mRNA vaccine production easier and shorter. However, the optimization of coding sequence, screening of modified nucleotides, and optimization of the delivery system are strategies to overcome the instability and elevation of translational efficiency of the mRNA vaccine (Silveira et al., 2021). These vaccines can be injected in various formulations such as carrier-mediated (encapsulated within lipid nanoparticles, polymers, and peptides), naked, and dendritic cell-based forms (Zeng et al., 2020).

Inactivated SARS-CoV-2 vaccines are used for the prevention of COVID-19. They are inactivated viruses which made through the growth of SARS-CoV-2 in the cell culture and then inactivation of the virus. There are different procedures of inactivation, such as the use of ultraviolet, gamma rays, glutaraldehyde, and formaldehyde. These vaccines are given intramuscularly and immune responses are induced against the nucleoprotein, envelope, matrix, and spike proteins (Krammer, 2020; Khoshnood et al., 2022). Ease of use, high speed of development, can be stored at 2–8°C, are the profits of using inactivated vaccines (Al Kaabi et al., 2021; Awadasseid et al., 2021).

Recombinant protein-based SARS-CoV-2 vaccines, also called recombinant subunit vaccines, contain only the antigenic components that are necessary to produce effective immune responses. Most of them focusing on the virus’s spike protein (Heidary et al., 2022). The advantage of using this proven approach in a pandemic situation is the ability to make a safe vaccine with a highly productive process that can be manufactured in existing facilities (Sartorius, 2022).

Other types of vaccine are live vector-based vaccines, which were introduced as second-generation vaccines. In these vaccines a genetically modified virus, as a vector, is used to express major exogenous protective antigen (s), use the host translational machinery, and then produce high-titer neutralizing antibodies. Live virus vectors function as an adjuvant to increase immunogenicity and induce a robust cytotoxic T lymphocyte (CTL) response to eliminate virus-infected cells (Ura et al., 2014). They generally are classified into two categories: replicating viral vectors that can propagate themselves in host cells and carry severe acute respiratory syndrome coronavirus 2 (SARS-CoV-2) proteins on its surface and nonreplicating viral vectors that also produce real virus proteins but fail to multiply owing to the disability of key genes; most of these vectors are based on adenoviruses (Ndwandwe and Wiysonge, 2021). The major limitations of some of the viral vectors entails low efficiency due to the pre-existence of antibodies, impossible use in immunocompromised patients, variable immunogenicity, chromosomal mutagenesis, and significant barriers for large-scale production and long-term storage (Strizova et al., 2021). Adenovirus (the causative agent of common cold), measles virus, and vaccinia virus are instances of viral vectors. Among all the vaccines designed, only a few have been approved. However, a great deal of effort is still ongoing to develop an effective vaccine against the SARS-CoV-2 virus. Herein, we provide a comprehensive review of the current knowledge, as well as the clinical developmental stage and use of nucleic acid vaccines against COVID-19.

## DNA vaccine candidates

**Figure 2:**
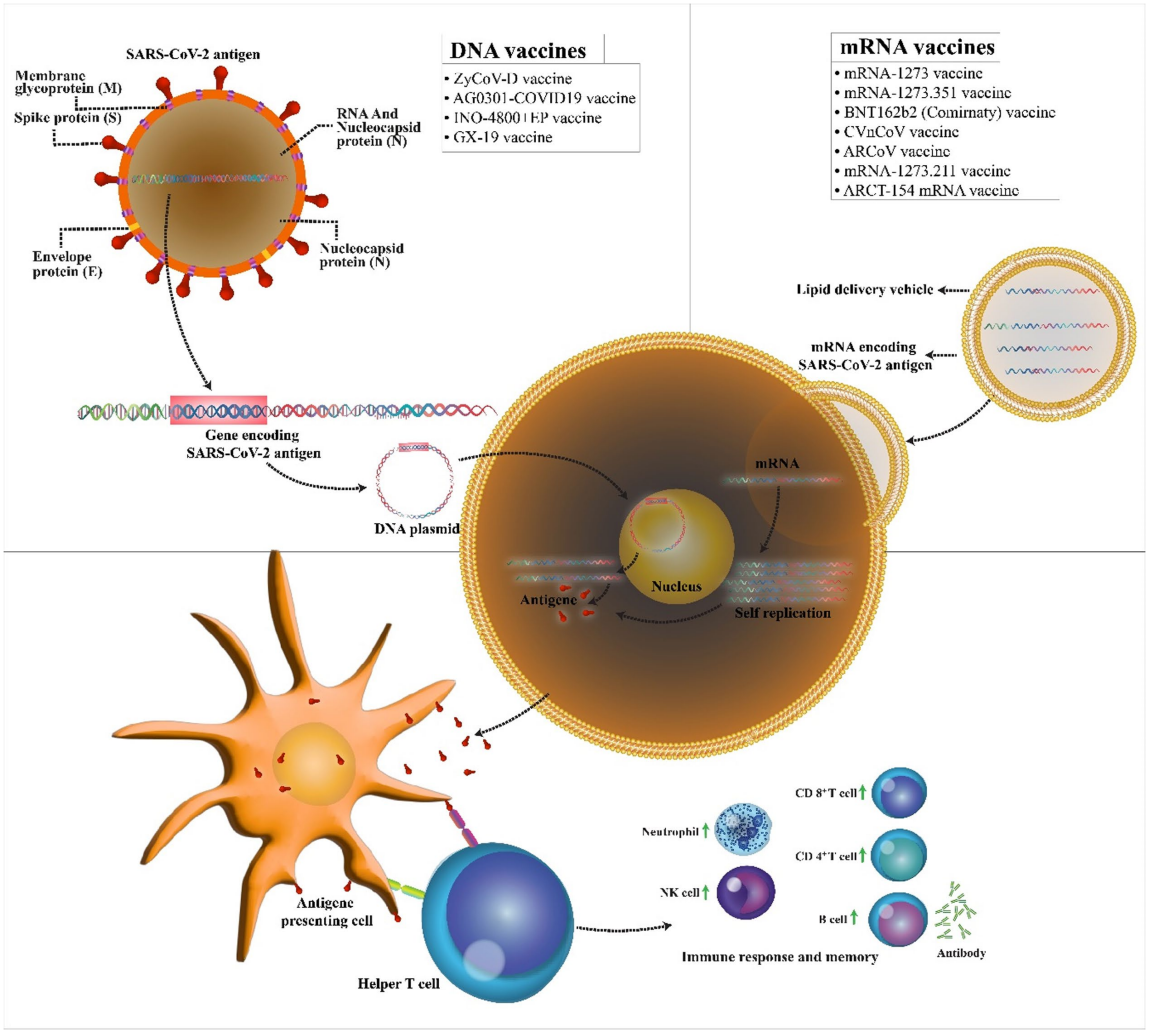
Mechanism of action of the COVID-19 DNA and RNA vaccines. COVID-19 mRNA vaccine has strands of messenger RNA inside a coating that protects it from enzymes. COVID-19 DNA vaccine comprises a plasmid that after the vaccine reaches the nucleus, it must be transcripted to an mRNA. In both vaccines, mRNA is translated into the human cells. Next, the encoded protein is expressed in the host cells, and the SARS-CoV-2 antigen can be then presented to antigen-presenting cells. Then, T helper cells stimulate the immune responses including an increase in neutrophils, NK cells, CD 4+ T cells, CD 8+ T cells, and B cells.

The possibility of easy development of DNA vaccine makes it an attractive option against rapidly emerging diseases like COVID-19. COVID-19 DNA vaccines (Table 1) are plasmids, including the gene of S protein, that are delivered into the body. After delivering, DNA will be converted to mRNA, and finally, the S proteins are produced. This process will cause cellular and humoral immunity and prevent the disease in infected patients (Figure 2).

**Table 1:** Characteristics of COVID-19 DNA vaccines.

Vaccine	Developer	Route of administration/ dose	Clinical stage	Subunit and structure	Adjuvant	Efficacy	Side effects	Reference
ZyCoV-D vaccine	Zydus Cadila; Department of Biotechnology, Government of India	IM/3	Phase 3	Plasmid DNA Antigen: non-specified	NR	66.6%	Negligible side-effects only in phase 1 (Data from efficacy trials had not been published)	Khobragade et al., 2022
AG0301-COVID19 vaccine	AnGes, Osaka University, Japan Agency for Medical Research and Development	IM/2	Phase1/2	Plasmid DNA Antigen: non-specified	Alum	NR (ongoing)	Not determinate	Chauhan et al., 2021
INO-4800 + EP vaccine	Inovio Pharmaceuticals/ International Vaccine Institute	ID (EP)/2	Phase2/3	PGX0001 expression vector Antigen: S protein	NR	100% (based on phase1 in the US)	No serious adverse events were reported (phase1: Erythema, pain, nausea, any local event).	Sanicas et al., 2020
GX-19 vaccine	Genexine Consortium in the Republic of Korea	IM (EP or PharmaJet® Needle-Free) /2	Phase2/3	Plasmid DNA Antigen: S protein	NR	NR (ongoing)	Phase 1(injection site pain, itching, tenderness, and fatigue)	Ahn et al., 2022

NR: Not Reported. IM: Intramuscular. IN: Intranasal. LNP: lipid nanoparticle.

The 4 of 11 candidate DNA vaccines were under phase 3 clinical trials to assess their safety and efficacy, however only one vaccine of them (ZyCoV-D) had approved from India. Efficiency ZyCoV-D vaccine was 66.6% against COVID-19 after two doses, without severe COVID-19-related side effect.

### ZyCoV-D vaccine

ZyCoV-D is the world’s first plasmid DNA vaccine for COVID-19; it got approval for Emergency Use Authorisation (EUA) from the Drug Controller General of India (DCGI) for healthy volunteers aged 12 years or older. ZyCoV-D is nonreplicating and nonintegrating plasmid DNA developed by Indian pharmaceutical firm Zydus Cadila with support from the Biotechnology Industry Research Assistance Council (Motamedi et al., 2021). Plasmid DNA vector carries genes encoding the spike protein of SARS-CoV-2, together with a promoter sequence that turns on the gene making the vaccine very safe. This candidate has a lab-made structure and is unable to interfere with the genetic composition of humans. It is a three-dose vaccine (three doses of 2 mg each) which is administered using a spring-powered jet injector, a needle-free applicator for painless intradermal vaccine delivery and reduces the any side effects and soreness at the site of vaccination. This vaccine has only side effects after the first dose of vaccine but not in another dose, so it could be a safe booster. After the plasmid uptake by the cell host, the encoded protein induces strong immunity, degrading during the time. Studies from animal trials have shown that it can elicit the protective level of neutralizing antibodies (Dey et al., 2021). Phase 1 clinical trial (CTRI/2020/07/026352) of ZyCoV-D, manufactured by India-based Zydus Cadila, was launched on 15th July 2020 and performed on 1,048 healthy volunteers with 18–55 years, to evaluate toleration and safety. Phase 2 study of the vaccine was conducted with more than 1,000 healthy adult volunteers in August 6, 2020 as a part of phase 1/2. Another phase 1/2 (CTRI/2021/03/032051) study was conducted to evaluate the safety and immunogenicity of two doses of 3 mg of the vaccine in 150 healthy volunteers aged 18–60 years. Considering the success of phase 1/2, the candidate vaccines entered phase 3 clinical trial (CTRI/2021/01/030416). This phase was a metacentric, randomized, double-blind, placebo-controlled trial carried out with three doses of the vaccine on 28,216 volunteers in the age group of 12–99 at days 0, 28, and 56 since November 2020. About 1,000 of these volunteers were in the age group of 12–17 years. In April 2022, Khobragade et al. demonstrated ZyCoV-D efficacy, safety, and immunogenicity in a phase 3 study interim analysis (Khobragade et al., 2022). Based on phase 3 clinical trial, the vaccine efficacy (VE) against symptomatic COVID-19 in volunteers vaccinated was 66.6%. Lower reactogenicity of ZyCoV-D vaccine rather than other currently used vaccines in India, the ease of manufacturing the vaccine with minimal biosafety requirements (BSL-1), fast adaptation to viral mutations, vaccine stability, and no cold chain for storage are advantages of this platform (Chary et al., 2020; Chauhan et al., 2021; Motamedi et al., 2021).

### AG0301-COVID-19 vaccine

AG0301/AG0302 COVID19 is a plasmid DNA expressing S protein antigen plus an adjuvant developed by AnGes Inc. (Osaka, Japan) in partnership with Osaka University (Chauhan et al., 2021). Antibodies produced against S protein blocked the binding of the virus spike to the cell’s receptor. It is a two-dose vaccine (first low then high dose at 2 weeks intervals) administered through the intramuscular. It is a non-randomized, open-label, non-controlled phase 1/2 study (NCT04463472) performed in June 2020 to assess the safety and immunogenicity of the vaccine in 30 healthy volunteers aged 20–65. All subjects under study were divided into the low (1.0 mg; *n* = 15) and high dose (2.0 mg; *n* = 15) groups. A phase II/III study (NCT04655625) was planned to evaluate the safety, immunogenicity, and efficacy of AG0302-COVID19 in healthy adult volunteers. In phase II/III, approximately 500 adult volunteers (18 years and older) were vaccinated intramuscularly with two doses of 2 mg at different interval levels [group A (*n* = 250): 2-week intervals, group B (*n* = 250): 4-week intervals]. Data from efficacy trials yet had not been published.

### INO-4800 + EP vaccine

A clinical trial of DNA vaccines has been initiated by INOVIO Pharmaceuticals (Pennsylvania, United States) and its partner Beijing Advaccine Biotechnology, with the support of the Coalition for Epidemic Preparedness Innovations (CEPI), Norway. INOVIO was the first company to start phase 2a vaccine for a related coronavirus that causes Middle East Respiratory Syndrome (MERS; Ye et al., 2020). INO-4800 is composed of a plasmid, namely pGX9501, designed to encode for the full length of the spike glycoprotein of SARS-CoV-2, the main coronavirus surface antigen with a leader sequence of IgE (Smith et al., 2020). This candidate injects DNA plasmids intradermally in 2-dose series by EP using the CELLECTRA 2000 device (Sanicas et al., 2020). This application improved the absorption of the antigen, thereby enhancing the immune response. Moreover, plasmids replicated into cells and induced natural response mechanisms in the host. A non-randomized open-label phase 1 trial (NCT04336410) of INO-4800 vaccine was started to investigate the safety, tolerability, and immunogenicity of the vaccine on April 3, 2020, in 40 healthy volunteers (18–50 years) in Philadelphia. The vaccine will be injected intradermally with two doses of 1–2 mg through EP within 4 weeks (Tebas et al., 2021).

Phase 1/2 trial (NCT04447781) was planned by the International Vaccine Institute (IVI) in collaboration with the Korean National Institute of Health (KNIH) to occur in South Korea concurrently with the US on 25 June 2020. This trial evaluated the safety, tolerability, and immunogenicity of 160 participants aged 19–64 years of age in two different parts (part A: open-label and part B: double-blind) in the Republic of Korea. Following potential safety and immunogenicity in phase 1/2 trial, phase 2 (randomized, double-blinded, placebo-controlled) clinical trial (ChiCTR2000040146) was performed to assess the immunogenicity, tolerability, and safety of the intradermal injection of different dose levels of the vaccine in 604 participants (18–50, 51–64, and 65 years and older) in China. Phase 2/3 (NCT04642638) of INOVIO Pharmaceuticals, namely INNOVATE (INOVIO INO-4800 Vaccine Trial for Efficacy), examined the safety and efficacy of two doses of 2 mg INO-4800 vaccine based on the data from the phase 2 evaluation in 6578 participants (≥18 years old) in the United States. This DNA vaccines are not currently on the market for use in humans (Funk et al., 2020). No treatment-related serious adverse events (SAE), type of adjuvant used, and efficacy assessment of this vaccine have been reported. Also, it has not received any approval.

### GX-19 vaccine

GX-19 is a DNA vaccine candidate that contained a 1:2 mix ratio of GX-19 (1 mg) and GX-21 (2 mg) developed by Genexine Consortium in the Republic of Korea and administered interadermally in two doses (day 1 and day 2). GX-19 is different from other COVID-19 vaccines: (Zhu et al., 2020) unlike other vaccines, Gx-19 targets both spike and nucleocapsid proteins, thereby inducing a wide range of antigen-specific T-cell responses and releasing a higher level of neutralizing antibodies that last longer in the body; (WHO, 2022) it targets parts of the virus that are shown to rarely mutate and provides protection against various variants of the COVID-19 virus; (Chavda et al., 2021) it delivers vaccine without adjuvant or viral vectors; hence, it can be safe for compromised adults and those with weakened immune system; (Abadi et al., 2018) the vaccine is delivered using a PharmaJet’s needle-free injection system that delivers medicine directly into muscle cells (Bahador et al., 2013).

In two studies of phase 1/2, clinical trials were evaluated in 170 (NCT04715997) and 210 (NCT04445389) healthy volunteers with 18–55 years of age, and the results indicated safety, tolerability, and immunogenicity in healthy volunteers (Chang et al., 2013; Clark et al., 2020). On July 9, 2021, a phase 2b/3 clinical trial was conducted on at least 14,000 healthy individuals (18 years and older) who have received one of the COVID-19 vaccines authorized for emergency use in multicountries. So far, 7 trials have been assessed safety and immunization in one country (India). The interim results for safety and efficacy were reported at the end of 2021 (DeJesus et al., 2004).

## RNA vaccine candidates

As the name implies, genetically engineered mRNA is used in the structure of RNA vaccines. After the vaccine is injected, the mRNA begins to produce the SARS-CoV-2 S protein on the surface of the person’s muscle cells, leading to antibody release. These antibodies will prevent COVID-19, if the person becomes infected with the SARS-CoV-2.

There are seven mRNA vaccines under phase 3 clinical trials to assess their safety and efficacy, however only two vaccines of them (*BNT162b2 (Comirnaty)*, and *mRNA-1,273*) had approved. The mRNA-1,273, and BNT162b2 vaccine effectiveness against infection of COVID-19 were 94.1, and 94%, respectively. There was anaphylaxis in 2.5 cases/ million doses of Moderna vaccine and 4.7 cases/million doses of Pfizer/BioNTech vaccine according to the Vaccine Adverse Event Reporting System (VAERS), followed by excess incidence of myocarditis/pericarditis particularly in younger individuals reported. Among all vaccine recipients, less than 2% serious systemic events (Pain, Swelling Redness, Allergy, Paralysis, Chills, Tiredness, Headache) were reported.

### mRNA-1273 vaccine

MRNA-1273 is a lipid nanoparticle (LNP)-encapsulated mRNA-based vaccine developed by Moderna and the Vaccine Research Center at the National Institute of Allergy and Infectious Diseases (NIAID; Table 2). In animal challenge experiments, the mRNA-1,273 vaccine has indicated protection, and in early-stage human testing, it has caused safety and immunogenicity (Corbett et al., 2020; Jackson et al., 2020).

**Table 2:** Characteristics of COVID-19 RNA vaccines.

**Vaccine**	**Developer**	**Route of administration/ dose**	**Clinical stage**	**Subunit and structure**	**Adjuvant**	**Efficacy**	**Side effects**	**Reference**
mRNA-1273 vaccine	ModernaTX, Inc.	IM/2	Phase 4	LNP	No adjuvant	100% efficacy against B.1.1.7; 95.7% efficacy against B.1.35 in Qatar and 94.1%in United States	Systemic adverse events were more common after the second vaccination. Serious adverse events were rare.	Baden et al., 2021; Chemaitelly et al., 2021; Corbett et al., 2021
mRNA-1273.351 vaccine	ModernaTX, Inc.	IM/2	Phase 2	LNP	No adjuvant	NR	NR	Baden et al., 2021
BNT162b2 (Comirnaty) vaccine	Pfizer-BioNTech	IM/2	Phase 4	LNP	No adjuvant	95% (based on phase 2/3)	Mild-to-moderate pain at the injection site within 7 days after injection, injection-site redness or swelling, fever, fatigue, and headache	Sahin et al., 2021
CVnCoV vaccine	CureVac N.V.	IM/2	Phase 3	LNP	No adjuvant	48.2% (phase 2b/3)	Injection-site pain, fatigue, headache, None of the fatal serious adverse events reported	Hoffmann et al., 2021; Kremsner et al., 2021
ARCoV vaccine	AMS, Walvax Biotechnology and Suzhou Abogen Bio-sciences	IM/2	Phase 3	LNP	No adjuvant	NR	NR	ClinicalTrials.gov, 2021a
mRNA-1273.211 vaccine	ModernaTX, Inc.	IM/2	Phase 2	LNP	No adjuvant	NR	NR	Clinicaltrials.gov, 2022b
ARCT-154 mRNA vaccine	Arcturus Therapeutic	IM/2	Phase1/2/ 3	self-amplifying mRNA	No adjuvant	NR	NR	Vietnam News Agency, n.d.; Clinical Trials Arena, 2021; ClinicalTrials.gov, 2022c

NR: Not Reported. IM: Intramuscular. IN: Intranasal. LNP: lipid nanoparticle.

In phase 3 placebo-controlled trial study, the efficacy of the vaccine was demonstrated to be the same across secondary analyses, i.e., assessment 2 weeks after the first dose, as well as in participants with the evidence of SARS-CoV-2 infection at baseline and in those who had 65 years of age or older. Severe COVID-19 was observed in all cases in the placebo group, with only one fatality. In the group vaccinated with mRNA-1,273, moderate and temporary reactogenicity was occurred. The efficacy of mRNA-1,273 vaccine in the prevention of symptomatic SARS-CoV-2 infection was greater than that observed in vaccines for respiratory viruses (Baden et al., 2021).

Recently, Moderna has provided preliminary data from phase 2/3 study of 30,000 cases more than 18 years of age in the US (Investors, 2020). In the study, 37% of subjects were from diverse racial/ethnic minority backgrounds, and 42% were subjects who were at high risk for severe COVID-19. Moderna emphasized that mRNA-1,273 vaccine was well tolerated across all populations with mild temporary adverse events and has an effectiveness of 94.5%, 2 weeks after the second dose. Five cases of COVID-19 were identified in the vaccine group and 90 cases in the placebo group; 15.8% of the COVID-19 cases were adults over 65 years of age, and 21.1% were from diverse racial/ethnic minority backgrounds. Additionally, 11 cases of severe COVID-19 were found in the placebo group, but in the vaccine group, no case was observed (Tumban, 2021). Another study investigated immune responses to NHP to different doses of mRNA-1,273 (Moderna) and evaluated that circulating spike protein-specific antibodies have a link to protection against SARS-CoV-2 replication in the airways. In hamsters, NHP antibodies transferred passively could mediate protection against SARS-CoV-2 challenge. Protection of the lower respiratory tract needs lower concentrations of serum antibodies, likely describing the reason the newest vaccines are highly effectual against severe lower airway disease. The higher antibody threshold necessary for the reduction of upper airway infection has the potential for increasing, in order to restrict transmission (Corbett et al., 2021). The efficacy of this vaccine has been reported in various studies of 100% efficacy against B.1.1.7 (Alpha); 95.7% efficacy against B.1.35(Beta) in Qatar (Chemaitelly et al., 2021) and 94.1% against wild type in United States (Baden et al., 2021). On January 31, 2022, the U.S. FDA approved this vaccine only to individuals above the age of 18 years and is not yet approved for children. This company is testing its efficacy for children ages 5–11.

### mRNA-1273.351 vaccine

During the global pandemic coronavirus disease, numerous mRNA-based vaccines were developed and distributed at an unprecedented rate to combat COVID-19, because of their high effectiveness against SARS-CoV-2. Moderna, by sponsoring the NIAID, introduced mRNA-1273.351 as a novel variant booster candidate against the variant B.1.351 initially identified in the Republic of South Africa on January 25, 2021. The booster vaccine was entered preclinical and phase 1 and 2 trials in the US by the company to assess the immunological effect of elevating strain-specific spike proteins (Chaudhary et al., 2021).

The administration of mRNA-1273.351 vaccine was carried out in immunization regimens alone or in combination with mRNA-1,273. LNP-encapsulated mRNA vaccine mRNA-1273.351 has the ability to encode a full-length, perfusion stabilized S protein of the SARS-CoV-2 variants of concern (VOC) B.1.351 (Clinicaltrials.gov, 2022b).

In an *in vivo* research by Moderna, the vaccine was examined as a two-dose primary series in mice and as a booster dose in animals formerly vaccinated with two doses of mRNA-1,273. Their observations revealed the effectiveness of a primary immunization series in boosting neutralizing antibody titers against the B.1.351 variant. Moreover, the third dosage of the vaccine raised neutralization titers in the wild-type D614G and B.1.351. These findings suggested the immunogenicity of the vaccine in mice. Later, Moderna displayed the preliminary outcomes of a clinical investigation on using mRNA-1273.351 vaccine almost 6 months earlier and concluded that the vaccine had an acceptable safety profile and was immunogenic (Wu et al., 2021a).

Wu et al., using SARS-CoV-2 neutralization assay, measured antibody neutralization titers against B.1.351 and P.1 variants before booster vaccination, i.e., 6–8 months after the primary dose. Their results showed low titers or below the assay limit of quantification, but higher titers against the wild-type strain. Following the initial immunization 2 weeks later, antibody titers against the two mentioned variants grew to levels similar to or higher than peak titers. In general, the efficiency of mRNA-1273.351 in elevating neutralization of the B.1.351 virus was more than mRNA-1,273. In phase 2 trial (Wu et al., 2021a).

In phase 2 study (Wu et al., 2021b), among 14,711 cases were given two primary doses of mRNA-1,273. In phase 3 study, cases were administered a single booster dose of mRNA-1273.351, which induced strong enhanced responses. Following the primary dose of the vaccine, neutralizing antibody titer against D614G virus was up to 4.4-fold higher than the peak titer. In the previously reported phase 2 and 3 studies, the safety profiles after mRNA-1273.351 and mRNA-1273 primary series were the same (Khoury et al., 2021; Madhi et al., 2021). The results suggested that variant-specific vaccines may increase former immune responses instead of transmitting novel immune responses to the variant epitopes (Otto et al., 2021).

In a study conducted by Ying and colleagues on 129S2 and K18-hACE2 mice, the immunogenicity and protective activity of mRNA-1273.351 vaccine were assessed. Immunization with high or low doses of the mRNA-1273.351 vaccine produced neutralizing antibodies against both SARS-CoV-2 and various variants. High dose formulation of the vaccine against all viruses induced protection from weight loss and lung pathology, while low dose indicated advanced lung infection and pneumonia with B.1.617.2. Therefore, due to the decreased levels of immunity by mRNA vaccines, the progress of infection and disease seems to happen with a number of SARS-CoV-2 variants, which emphasizes the need for further booster regimens (Ying et al., 2021). The available vaccines have exhibited partial or complete effectiveness against the original use of SARS-CoV-2 and its variations, which should be promoted and extended for broad vaccination.

This is a phase 1, open-label, randomized clinical trial in males and non-pregnant females, 18 years of age and older, who are in good health, have no known history of COVID-19 or SARS-CoV-2 infection, and meet all other eligibility criteria. This clinical trial is designed to assess the safety, reactogenicity and immunogenicity of mRNA-1273.351 manufactured by ModernaTX, Inc., given in vaccination schedules alone, sequentially, or coadministered with mRNA-1,273.

### BNT162b2 (comirnaty) vaccine

Comirnaty is a nucleoside-modified RNA vaccine approved by the US Food and Drug Administration (Lustig et al., 2021). Pfizer Biontech COVID-19 vaccine encodes a prefusion-stabilized SARS-CoV-2 spike protein encapsulated in a LNP to elevate its uptake by host immune cells (Granados-Riveron and Aquino-Jarquin, 2021).

Based on preclinical findings, the immunization of NHPs (rhesus macaques) with BNT162b2 led to strong antiviral impacts on SARS-CoV-2 infection. In all SARS-CoV-2-challenged rhesus macaques, vaccination with this vaccine inhibited lung infection, and no viral RNA was observed in the lower respiratory tracts of the vaccinated and challenged animals. BNT162b2 immunization, within 3 days after infection, cleared the viral RNA in all SARS-CoV-2-challenged animals. The vaccine produced SARS-CoV-2 and pseudovirus-neutralizing antibodies in rhesus macaques and mice, respectively, as well as induced potent antigen-specific CD4^+^ and CD8^+^ T cells in both animal groups (Pfizer, 2020).

Based on the phase 1/2/3 study, immunization with BNT162b2 at well-tolerated doses produced a mixed adaptive humoral and cellular immune response, which possibly contributes to protection from COVID-19 (Sahin et al., 2021). Emerging evidence has also indicated that the effectiveness of the mRNA-based vaccine against the original SARS-CoV-2 and the B.1.1.7 variant was 95% (Lustig et al., 2021), which supports the findings of Polack and associates who reported 95% effectiveness of the vaccine in avoiding COVID-19. The efficacy of BNT162b2 was the same across subgroups identified by gender, age, ethnicity, race, baseline BMI, and the presence of concomitant diseases. The safety profile of the vaccine included short-term, mild-to-moderate pain at the site of injection, fatigue, and headache (Polack et al., 2020).

In an ongoing placebo-controlled trial, 2,260 participants, aged 12–15 years, received injections; 1,131 cases were administered BNT162b2, while 1,129 cases were given placebo. In other age groups, the vaccine showed side effects, but with temporary mild-to-moderate reactogenicity. The vaccine also induced a higher immune response than in young adults. The overall efficacy of the vaccine was found to be 100% (Frenck Jr et al., 2021). In phase 2 controlled experiment, BNT162b2 vaccine was administered as a second dose to individuals who were vaccinated with ChAdOx1-S. Vaccination induced a strong immune response with an acceptable and controllable reactogenicity (Borobia et al., 2021).

Collectively, the BNT162b2 vaccine showed superb effectiveness and safety in phase 3 and robust immunogenicity in phase 2 studies. In phase 4 trial, titers of neutralizing IgG were assessed against the RBD of the S1 subunit of the SARS-CoV-2 spike protein 2 weeks after vaccination of patients. Age-related antibody titers displayed no significant difference in the range of 20–50 years, but in the 50–60 age group, antibody titers were significantly lower (Kontou et al., 2021). The Phase 1/2/3 trial, safety, tolerability, and immunogenicity of the Pfizer-BioNTech COVID-19 Vaccine evaluated among 10,000 children (aged 6 months–12 years) in the United States, Finland, Poland, Spain and Brazil. This vaccine approved by FDA under Emergency Use Authorization (EUA) to prevent of covid-19 infection in subjects 6 months of age and older. 87 trials in 27 countries assess safety and immunization of this vaccine. This vaccine approved in 146 countries.

### CVnCoV vaccine

CVnCoV, designed by CureVac N.V. (Germany) and the Coalition for Epidemic Preparedness Innovations (Norway), is a LNP vaccine encoding perfusion stabilized spike protein. CVnCoV vaccine, unlike other mRNA-based vaccines, comprises of non-chemically altered nucleotides and can be used at low doses. In NHPs, CVnCoV can induce robust humoral and cellular responses, and animals given 8 μg of the vaccine can be protected from infection with SARS-CoV-2. Studies of pathological alterations in challenged animals gave no evidence of increased. Disease after vaccination with CVnCoV, which is suggestive of the safety, immunogenicity, and protective effectiveness of the vaccine (Rauch et al., 2020).

A former study has highlighted that immunization with CVnCoV vaccine could induce robust humoral responses with high titers of antibodies neutralizing the virus and strong T-cell responses. Vaccination of hamsters with CVnCoV made a protection against challenge with wild-type SARS-CoV-2, but those vaccinated with a minimal dose of the vaccine showed no indication of increased disease due to vaccine. Altogether, CVnCoV was recognized to be a strong and safe vaccine candidate against SARS-CoV-2 (Rauch et al., 2021).

According to data from a phase 2b/3 trial of CVnCoV, the vaccine designed for the completion of phase 3 trials had only 47% effectiveness against the disease, which contradicts previous mRNA vaccines developed by Moderna and Pfizer BioNtec that showed almost twice efficacy (Baker and Dolgin, 2021). The correlation of neutralizing antibody response with protection against SARS-CoV-2 infection was analyzed across eight vaccine platforms (Cromer et al., 2021). One study, using the K18-hACE2 transgenic mouse model, tested the effectiveness of CVnCoV against the ancestral strain and VOC B.1.351. Based on the results, the vaccine induced full protection against both disease and mortality. Notwithstanding reduced neutralizing antibody titers in comparison to BavPat1 as the ancestral strain, CVnCoV displayed full disease protection (Hoffmann et al., 2021). In another study, a dosage escalation phase 1 was analyzed in healthy subjects with the age of 18–60 years old; those who received two CVnCoV vaccinations or placebo displayed no serious AEs related to vaccine. In general, two doses of CVnCoV vaccine showed to be safe and had reactogenicity (Kremsner et al., 2021). A survey in Poland evaluated eight unauthorized vaccines in the EU. The vaccine with the highest level of trust belonged to CVnCoV, followed by Ad26.COV2.S, NVX-CoV2373, and Sputnik V (Rzymski et al., 2021). Phase 2b/3 study show that the efficacy of this vaccine is 48.2%. The most frequently reported local reaction was injection-site pain, and the most frequently reported systematic reactions were fatigue and headache. 0·4% recipients reported 100 serious adverse events. None of the fatal serious adverse events reported were considered to be related to study vaccination (Kremsner et al., 2022).

### ARCoV vaccine

The Arabic vaccine, ARCoV or Walvax COVID-19 vaccine, is an mRNA vaccine developed with relying on the RBD of SARS-CoV-2 (Park et al., 2021). The vaccine was first designed by the collaboration of Walvax Biotechnology with the Academy of Military Medical Sciences and Suzhou Abogen Biosciences Co., Ltd. (China). Intramuscular immunization of ARCoV mRNA LNP in mice and NHPs induced strong neutralizing antibodies to SARS-CoV-2 and to a Th1-biased cellular response. Immunization of mice with two doses of ARCoV elicited full protection against SARS-CoV-2. ARCoV vaccine, which is produced as a liquid formulation, can be preserved at room temperature for minimum 1 week. The safety, tolerance, and preliminary immunogenicity of this vaccine assessed in phase 1 clinical trials (Zhang et al., 2020). Fever and Low lymphocyte count were most common adverse reaction. In January 2022, Phase II trial was conduct on 420 participants aged 18–59 to evaluate immunogenicity and safety of different doses. Phase 3 Clinical studies of this vaccine were conducted in 28,000 people in Mexico, Indonesia, Nepal, and China (ClinicalTrials.gov, 2021a).

### mRNA-1273.211 vaccine

mRNA-1273.211 is an updated new mRNA booster vaccine stabilizing the perfusion form of spike protein (Genetic Engineering & Biotechnology News, 2022). The vaccine combines mRNA-1,273 and mRNA-1273.351 (Pharmaceutical Technology, 2021). Wu and associates conducted a study on mice and evaluated two novel COVID-19 mRNA vaccines developed for targeting SARS-CoV-2 variants. The first vaccine, mRNA-1273.351, is found in the B.1.351 lineage and encodes for the S protein, and mRNA-1273.211 is the second vaccine consisting of a mixture (1:1) of mRNA-1,273 and mRNA-1273.351. The two aforesaid vaccines were studied in mice as a two-dose initial series. The first vaccine was also assessed as a booster dose in animals vaccinated with two doses of mRNA-1,273. Their results evinced the effectiveness of both vaccines and emphasized that the third dose of mRNA-1273.351 could considerably elevate both wild-type and B.1.351-specific neutralization titers. The two vaccines are now being assessed in both preclinical challenge models and in phase 1/2 clinical trial (Wu et al., 2020).

In a clinical study, three parts, A, B, and C, were evaluated. In part A, two doses (50 or 100 μg, IM) of the mRNA-1273.211 vaccine, as a single booster dose, were given to the adults of the mRNA-1,273-P301 (COVE [NCT04470427]) study. These participants had formerly received two doses of mRNA-1,273. In parts B and C, the mRNA-1,273 and mRNA-1273.617.2 vaccines were, respectively, administered as a single booster dose (100 μg) to the same adults mentioned above. The enrollment of part B will initiate after completing the enrollment of part A and part C after the completion of the enrolment of part B of the study (ClinicalTrials.gov, 2022a). In another survey, Choi et al. evaluated the safety and immunogenicity of a single booster dose of mRNA-1,273, modified mRNA-1273.351, and multivalent mRNA-1273.211 in healthy adults in phase 2a trial. Their results demonstrated that all boosters were safe and well-tolerated and numerically enhanced neutralization titers against SARS-CoV-2 VOCs, B.1.351, P.1, and B.1.617.2. Their clinical trial study is still ongoing (Wu et al., 2021a). So far, 4 trials have been assessed safety and immunization in one country (U.S).

### ARCT-154 mRNA vaccine

ARCT-154 is a two-dose self-amplifying mRNA vaccine to be produced under the collaboration of VinBioCare (a member of Vingroup, the largest private conglomerate in Vietnam) and Arcturus (Clinical Trials Arena, 2021). The vaccine was developed based on the ARCT-021 vaccine, whose research results in phases 1, 2, and 3 have been reported in the US and Singapore. Given this, the Health Ministry’s Biomedical Ethics Council permitted phases 2 and 3a clinical trials to take place at the same time (Vietnam News Agency, n.d.). The two phases were carried out simultaneously in Bắc Ninh, Hanoi, and Long An with a total of 1,000 volunteers. The trial work was carried out by Hanoi Medical University and Pasteur Institute in Ho Chi Minh City. In Bắc Ninh, from 20 to 23 September, the research team started recruiting volunteers and selected 338 people aged 18–65, who received the first dose from 27 to 29 September. In Long An and Hanoi, the Ministry of Health also implemented the first dose for volunteers. The phase 3 trial is expected to end on November 24th 2021, and the research team will report the results of the trial to the Ministry of Health on December 30 (ClinicalTrials.gov, 2022c). The studies showed this vaccine achieved 55% efficacy against infection and 95.3% against severe disease.

## Viral vector (nonreplicating) vaccine candidates

**Figure 3:**
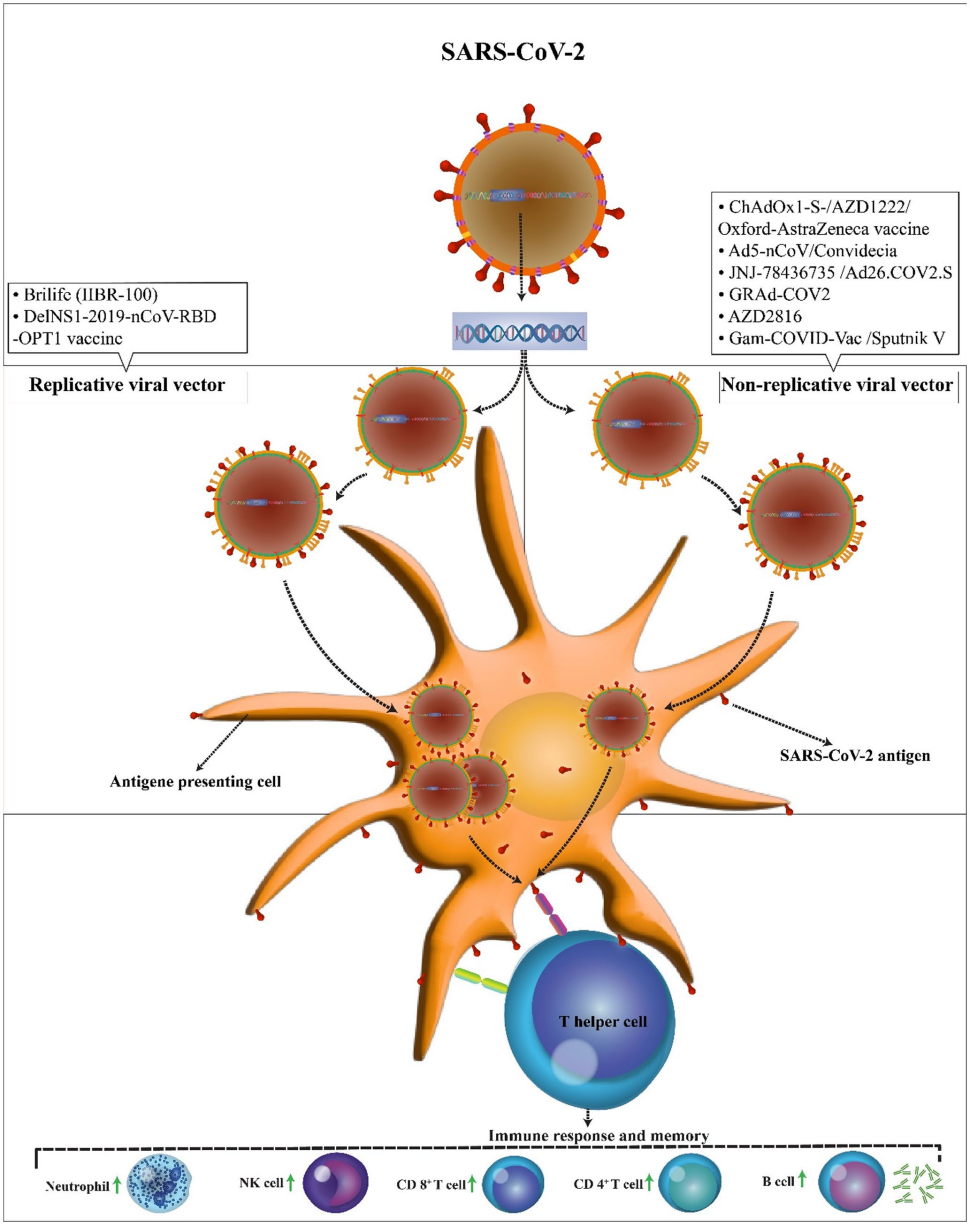
Mechanism of action of the COVID-19 vector vaccines. In comparison to replicating viral vectors, nonreplicating vectors have genetic deletions and unable to replicate inside the body. In both vaccines, the SARS-CoV-2 antigen can be presented to antigen-presenting cells. Then, T helper cells stimulate the immune responses including an increase in neutrophils, NK cells, CD 4+ T cells, CD 8+ T cells, and B cells.

In comparison to replicating viral vectors, nonreplicating vectors have genetic deletions. These deletions allow nonreplicating viral vectors to accept twice as larger genetic elements than replicating viral vectors (8 kb vs. 4 kb). However, as nonreplicating vectors are unable to replicate inside the body, much higher doses are needed to promote immune responses (Figure 3). There are six non-replicating viral vector vaccines under phase 3 clinical trials to assess their safety and efficacy, however only two vaccines of them (GRAd-COV2 vaccine and AZD2816 vaccine) yet not approved. The ChAdOx1-S, Ad5-nCoV, and Ad26.COV2.S vaccines received approval for emergency use by the United States Food and Drug for individuals of 18 years and above. ChAdOx1m, SPUTNIK V, Ad5-nCoV, Ad26.COV2 had an efficacy of 76, 97.6, 65.7, 66% at preventing symptomatic COVID-19 infection and 100,100, 90.98, 85% efficacy in preventing severe COVID-19 infection. The majority of the systemic adverse reactions in the clinical trials were mild to moderate intensity and no serious events. The common side effects were fever, headache, nausea, fatigue, and myalgia. The most of side effects reported in patients under the age of 60 years.

### ChAdOx1-S-(AZD1222) vaccine

The Oxford/AstraZeneca vaccine, AZD1222, also known as Vaxzevria in the EU and ChAdOx1 nCoV-19, is an adenovirus-based viral vector vaccine containing a genome encoding a perfusion conformation of the SARSCoV2-spike glycoprotein antige (Table 3; CDC, n.d.). In the past, viral vector vaccines using adenovirus have demonstrated difficulties with immunity to viral vectors, but there was not any indication of tumorigenicity (Kaur and Gupta, 2020). Immunization of BALB/c and CD1 mice, pigs, ferrets, and NHPs with AZD1222 has been exhibited to be immunogenic in these animals (Ramasamy et al., 2020).

**Table 3:** Characteristics of COVID-19 non-replicating viral vectors vaccines.

**Vaccine**	**Developer**	**Route of administration/ dose**	**Clinical stage**	**Subunit and structure**	**Adjuvant**	**Efficacy**	**Side effects**	**Reference**
ChAdOx1-S-/AZD1222/ Oxford-AstraZeneca vaccine	AstraZeneca and the University of Oxford	IM /2	Phase 4	Non-replicating viral vector (chimpanzee adenovirus vector encoding the SARS-CoV-2 Spike protein)	No adjuvant	66.7% based on results of four clinical trials in the UK, Brazil, and South Africa	Fever, headache, muscle pain, numbness, eye muscle pain. thrombotic events (few studies)	Pormohammad et al., 2021; Voysey et al., 2021; Alghamdi et al., 2022
Ad5-nCoV/Convidecia	CanSino Biological Inc. and Beijing Institute of Biotechnology	IM /1	Phase 4	Non-replicating viral vector (adenovirus type 5 vector expressing the SARS-CoV-2 coronavirus spike protein)	No adjuvant	63.7 and 57.5% after day14 and 28 post-administration (according to phase3 data)	Commonly mild reaction: Headache, pain at the injection site	Halperin et al., 2022
JNJ-78436735/Ad26.COV2.S	Janssen Pharmaceutical Companies (Johnson & Johnson)	IM /1	Phase 4	Non-replicating viral vector (adenovirus type 26 expressing the SARS-CoV-2 spike protein)	No adjuvant	66.9% based on phase 3 clinical trial data	generally mild to moderate and temporary with a lower incidence among older subjects	Sadoff et al., 2022
GRAd-COV2	ReiThera, Leukocare and Universelle	IM /1	Phase 2/3	Non-replicating viral vector (gorilla Adenovirus encoding SARS-COV-2 Spike protein)	No adjuvant	98.8% seroconversion rate based on phase 1 data	mostly mild or moderate and with a short duration	Lanini et al., 2021b
AZD2816	AstraZeneca and the University of Oxford	IM /2	Phase 2/3	Non-replicating viral vector (similar to AZD1222; but encoding beta variant SARS-COV-2 Spike protein)	No adjuvant	NR	NR	van Doremalen et al., 2022
Gam-COVID-Vac /Sputnik V	Gamaleya Research Institute and Health Ministry of the Russian Federation	IM /2	Phase3	Non-replicating viral vector (adenovirus serotype 26 encoding SARS-COV-2 Spike protein)	No adjuvant	91·6% efficacy based on phase 3 data	pain in the injection site, fatigue, body pain, headache, fever, joint pain, chilling, and drowsiness(based on Iranian health care workers’ results)	Babamahmoodi et al., 2021; Logunov et al., 2021

NR: Not Reported. IM: Intramuscular. IN: Intranasal. LNP: lipid nanoparticle.

In phase 2/3 study, a single dose of the vaccine produced humoral and cellular immune responses in rhesus monkeys and induced protection against lower respiratory tract infection following subsequent infection with SARSCoV2.17 (Tian et al., 2021). Results from a phase 1/2 study in adults aged 18–55 years have displayed that the vaccine is well-tolerated and induces stable neutralizing antibodies and cellular immune responses to the spike glycoprotein (Folegatti et al., 2020). In one study, the efficacy of four trials (COV001, COV002, COV003, and COV005) was analyzed among 17,178 people. The study detected 332 cases of COVID-19 symptoms in more than 2 weeks following the second injection of the vaccine. Moreover, 84 positive cases and 248 cases were identified in the AZD1222 and control groups, respectively, reflecting an efficacy of 66.7%. At 3 months after the second injection, no signs of reduced efficacy were observed against symptomatic COVID-19 in relation to the vaccine (Voysey et al., 2021). The immunogenicity data for COV001 suggested that the second dose of the vaccine provided enhanced immunogenicity; therefore, a two-dose regimen was used (Lefebvre et al., 2021). In a randomized controlled trial with HIV-negative cases, the efficacy and safety of AZD1222 vaccine were assessed. Participants (aged ≥18 < 65) were randomly selected in a 1:1 ratio to receive two doses of the vaccine or placebo within 21–35 days. In this secondary trial evaluating the effectiveness of AZD1222 against VOE B.1351, 39 cases of the variant were reported. In addition, there was little to no evidence that AZD1222 protected against B.1351 variant, with a VE of 10.4% (Madhi et al., 2021).

The frequencies of side events reported for AZD1222 vaccine were similar to those of the AstraZeneca vaccines, and the majority of the adverse effects had no relationship with the vaccine. In phase 3 trials, 12,000 cases were given the AZD1222 vaccine; only one case of transverse myelitis was found to be associated with the vaccine (Lefebvre et al., 2021).

The more time passes, the more we know about severe adverse events occurred following AZD1222 vaccine administration. In addition to common and mild events, reports of thrombotic adverse reactions in some countries since March 2021, resulted in a temporary cession of vaccine usage (Belete, 2022).

A retrospective descriptive analysis based on EudraVigilance database on frequency of thrombotic adverse events among the population who received one of Moderna, Pfizer and Oxford-AstraZeneca vaccines showed 53.8% of these events were related to Oxford-AstraZeneca vaccine in the age group 18–64 years, but interestingly two other vaccines showed also high proportion among subjects in 65–84 years group, indicating almost equal prevalence of thrombotic adverse events between these vaccines that is relevant to the age group (Tobaiqy et al., 2021). 69 trials in 32 countries assess safety and immunization of this vaccine. Oxford-AstraZeneca vaccines approved in 141 countries. The studies showed this vaccine had an efficacy of 72% against symptomatic SARS-CoV-2 infection and 75% against Omicron virus variant. Also, WHO published interim recommendations for use this vaccine.

### Ad5-nCoV vaccine

Ad5-nCoV, with the trade name of Convidecia, is a genetically engineered vaccine developed in China and encodes the full-length SARS-CoV-2 spike protein. Vaccines designed based on the adenoviral vectors are easily developed and can be produced on a large scale, which provides a promising platform for clinical use. For instance, there are many human clinical trials performed for adenoviral vector-based vaccines against varied infectious diseases. The results of Ad5-nCoV studies have represented that these vaccines have ability to elicit pathogen-specific humoral and cellular immunity (Cao et al., 2021). In an animal trial, it has been explored that the vaccination of mice with a single dose of Ad5-nCoV induces full protection against mouse-adapted SARS-CoV-2 infection, both in the upper and lower respiratory tracts. The trial also highlighted that mucosal vaccination could provide a favorable protective efficacy (Wu et al., 2020).

With the aim of evaluating the safety and immunogenicity of the Ad5-nCoV vaccine, a phase 1 trial was conducted in Wuhan city of China. The trial included adults (aged ≥18 years) seronegative for SARS-CoV-2. The most frequent AD observed 7 days after the first booster vaccine was fever (in 48% of cases), followed by fatigue (31%) and headache (35%). Aerosolized Ad5-nCoV was well tolerated, and two doses of this vaccine induced neutralizing antibody responses the same as one dose of intramuscular injection. At 28 days after the first intramuscular injection, an aerosolized booster vaccination elicited potent IgG and neutralizing antibody responses (Wu et al., 2021c).

An important step to approve vaccines is the clinical assessment of the immune response, including immunogenicity, safety, and efficacy (Cao et al., 2021). Determination of the dose of the Ad5-nCoV vaccine was assessed in the first randomized controlled trial, comprising of 508 eligible subjects assigned to receive the vaccine (1 ×  10^11^ and 5 ×  10^10^ viral particles) or placebo. Both doses of Ad5-nCoV induced significant neutralizing antibody responses to live SARS-CoV-2. The Ad5-vectored COVID-19 vaccine showed to be safe at 5  ×  10^10^ viral particles and induced significant immune responses in most of the subjects after a single immunization (Zhu et al., 2020). In a survey conducted on 54 cases who received Ad5-nCoV vaccine, the immunogenicity of the messenger adenovirus vector Ad5-nCoV vaccine was discovered through the generation of IgG antibody to subunit 1 of protein S, and the side effects of the vaccine were evaluated. The vaccine induced S1 IgG antibodies in 88.89% of the vaccinated cases. Females induced more antibodies when receiving either vaccine. Ad5-ncov was concluded to be a safe vaccine (Guzmán-Martínez et al., 2021).

Phase 3 of the vaccine clinical trial with 36,717 participants aged 18 years and older conducted in Argentina, Chile, Mexico, Pakistan, and Russia from Sept 2020 to Jan 2021 to investigate immunogenicity and safety of single-dose Ad5-nCoV vaccine. The vaccine was well-tolerated and pain at the injection site was the most frequent side effect. Observation of severe adverse events was rare and similar (0.1%) in both vaccine and placebo groups. Efficacy of vaccine against symptomatic and PCR-confirmed COVID-19 infection at day14 and 28 after administration were 63.7 and 57.5%, respectively, (Halperin et al., 2022).

In a longitudinal investigation, 346 cases (117 with and 229 without prior COVID-19) who were vaccinated with Ad5-nCoV were selected. The vaccine induced higher neutralizing antibody percentage in individuals with (98%) than without (72%) prior COVID-19. Moreover, before vaccination, a natural infection induced more neutralizing antibodies than immunized people without prior COVID-19. None of the patients showed severe adverse effects related to the vaccine. Production of impaired antibodies was associated with age, antidepressant and immunosuppressive treatments, reactogenicity, and history of COVID-19. In all groups, after 21 days of post vaccination, the anti-Ad5 antibodies increased (Hernández-Bello et al., 2021).

The efficacy of Ad5-nCoV with one dose has been concerned. In an investigation, SARS-CoV-2 spike 1–2 IgG antibodies in plasma samples were compared between two groups. The first group was immunized with Ad5-nCoV vaccine, and the second group received a heterologous vaccination regimen containing Ad5-nCoV and BNT. Cases who received BNT as a boost after Ad5-nCoV showed higher SARS-CoV-2 spike 1–2 IgG antibody titers, without any severe adverse effect (Romero-Ibarguengoitia et al., 2021). 14 trials in 6 countries assess safety and immunization of this vaccine and was approved in 10 countries. In Jan. 11, 2022, a phase 1/2 study showed boosting dose with inhalation Convidecia induced a strong mucosal immune response. On May 19, 2022, the WHO published interim recommendations for use this vaccine.

### Ad26.COV2.S vaccine

On February 27, 2021, Johnson and Johnson’s vaccine, Ad26.COV2.S, was authorized for use in the US. Ad26.COV2.S has hitherto been administered to more than eight million patients. The vaccine is a recombinant, replication-incompetent human adenovirus type 26 vector. Ad26.COV2.S vaccine causes the expression of the SARS-CoV-2 spike antigen through mRNA or a viral vector, without propagating the virus. Protection against COVID-19 is occurred when the immune response is elicited to the S antigen (Sulemankhil et al., 2021).

In a preclinical experiment with NHPs, the immunogenicity and protective efficacy of a single dose of the vaccine were examined. After the SARS-CoV-2 challenge, the vaccine induced strong neutralizing antibody responses and made a complete or near-complete protection in bronchoalveolar lavage and nasal swabs. There was a connection between the titers of vaccine-elicited neutralizing antibodies and protective efficacy, which is the indication of an immune correlate of protection (Mercado et al., 2020).

Understanding the immune responses to SARS-CoV-2 contributes to the perception of disease pathogenesis and the usefulness of treatment, as well as assists in the development of vaccines, antivirals, and monoclonal antibodies (Sadoff et al., 2021). In a placebo-controlled, phase 1-2a trial, healthy individuals aged 18–55 years (cohort 1) and aged 65 years or older (cohort 3) were randomized to receive two doses of Ad26.COV2.S. After administrating the first dose of the vaccine, fever was the most common AV, and after the second dose, reactogenicity was lower. Following the first vaccine dose, titer neutralizing antibodies against wild-type virus were observed in the majority (90% or more) of all subjects on day 29 and reached 96% by day 57, with elevated titer in cohort 1a. However, the titers remained stable until at least day 71. Spike binding antibody response and neutralizing antibody response were found to be the same. On day 15, CD4^+^ T-cell responses were identified in 76–83% (in cohort 1) and in 60–67% (in cohort 3). CD8^+^ T-cell responses were strong, though was lower in cohort 3 (Sadoff et al., 2021). In an international, randomized, placebo-controlled phase 3 trial, adult subjects (in a 1:1 ratio) were randomly assigned to receive a single dose of Ad26.COV2.S vaccine (5 × 10^10^ viral particles) or placebo. On onset at least two and 4 weeks after administration, the vaccine protected against moderate to severe critical COVID-19, with the efficacy of 66.9%. A higher efficacy was also observed against severe critical COVID-19, with onset at ≥14 days (76.7%) and at ≥28 days (85.4%). Reactogenicity with the vaccine was higher than with placebo but was generally mild to moderate and temporary with lower incidence among older subjects. Safety was speculated to be comparable to that observed in other phase 3 trials of COVID-19 vaccines (Poland et al., 2020; Sadoff et al., 2022). 24 trials in 24 countries assess safety and immunization of this vaccine and was approved in 111 countries. Studies showed that this vaccine simulated robust cellular protection against serious infection caused by the highly transmissible Omicron (B.1.1.529), Alpha (B117) and Delta (B1617.2) variant circulation. The WHO published interim recommendations for use this vaccine.

### GRAd-COV2 vaccine

GRAd-COV2 was developed for the first time by ReiThera, LEUKOCARE, and Univercells. The vaccine uses GRAd32, a Gorilla Adenovirus belonging to the group C adenovirus family, to deliver an engineered SARS-CoV-2 spike protein sequence. A very recent study has shown that GRAd-COV2 vaccine is highly immunogenic, particularly in mice, and macaques can neutralize SARS-CoV-2 infection and inhibit the spike protein of SARS-CoV-2 binding to the ACE2 receptor. That study also demonstrated that the prefusion stabilized spike antigen is superior to the wild type in inducing ACE2-interfering, i.e., antibodies neutralizing SARS-CoV-2. Considering these data, GRAd-COV2 was recognized to be a potential COVID-19 vaccine candidate (Capone et al., 2021). Another investigation reported the safety and immunogenicity of GRAd-COV2 in healthy younger and older adults after receiving a single intramuscular dose of the vaccine at three escalating doses. To this end, a phase 1 trial was carried out and included 90 healthy people. All subjects were intramuscularly given a single dose of the vaccine at three increasing doses. Results of enzyme-linked immunosorbent assay (ELISA) revealed that 89 of 90 (98.8%) volunteers raised detectable anti–spike protein IgG at week 4 after vaccine injection. Furthermore, a robust T helper 1 cell-mediated cellular immunity response was detected against the spike protein in 89 of 90 participants. Almost all reported reactions were mild and short lived. The safety and immunogenicity data from phase 1 clinical trial affirmed the additional development of GRAd-COV2 vaccine (Lanini et al., 2021a,b).

In phase 2 trial (NCT04791423), almost 900 participants received one single intramuscular dose of both GRAd-COV2 (2 × 10^11^) and placebo after 21 days or two repeated (21 days apart) intramuscular dose of the vaccine (1 × 10^11^) or two doses of placebo on day 1 and day 22. Phase 3 trial will be adapted under the following circumstances: a superiority trial vs. placebo not only on the overall population but also on a subset of populations, as well as a noninferiority trial vs. the available alternative vaccine(s) on a surrogate endpoint, if available (ClinicalTrials.gov, 2021b). So far, 5 trials in 12 countries assess safety and immunization of this vaccine.

### AZD2816 vaccine

AZD2816 vaccine was developed by Astra Zeneca and collaborators using the adenoviral vector platform similar to Vaxzevria, formally AZD1222, with minor genetic changes to the spike protein on the basis of the Beta COVID-19 variant, B.1.351. The Beta variant vaccine comprises of 10 alterations across the spike protein; most of which can be found in other variants. These changes often decrease the capability of antibodies induced against the original virus to inhibit cell entry, raise infectivity compared to the original virus, and decline the sensitivity of neutralizing antibodies to the original virus. Regardless of minor changes, the two vaccines are totally the same (AstraZeneca, 2021).

A survey conducted on the evaluation of a ChAdOx1-vectored vaccine against new SARS-CoV-2 virus isolates or variants of concern (VoC) B.1.351 explored the immunogenicity of AZD2816 vaccine following a single dose. The study also found that in mice primed with AZD1222 vaccine and then received AZD2816 vaccine as a booster dose, no indication of “original antigenic sin” was detected, instead, high titer antibodies were observed against a number of variant spike proteins including Gama, Beta and Delta variants (Spencer et al., 2021).

In another study similar to the previous one, the effectivity of AZD2816 single dose or as a booster for AZD1222 vaccine was challenged in hamsters were infected with deferent variants, also Omicron. On day5 after inoculation, there was minimal to no subgenomic viral RNA in lung tissue, reflecting the vaccine preventive effect against this new variant (van Doremalen et al., 2022). So far, 2 trials have been assessed in 2 countries.

### Sputnik V vaccine

Gam-COVID-Vac or Sputnik V was registered for the first time in the world by the Decree of the Government of the Russian Federation No. 441 dated 3 April 2020 (Naygovzina et al., 2021). Gam-COVID-Vac uses two (Ad26 priming and Ad5 boost) vectors to decrease the risk of reduced effectiveness of vaccination. The Sputnik V vaccine benefits from high efficiency (97%), low cost, and low cold storage temperatures (Cazzola et al., 2021).

Based on phase 3 clinical trial data, the efficacy of the vaccine was 91.6%. Grade 1 was the most reported AVs, and serious AVs were observed in 0.3 and 0.4% of participants. Moreover, four deaths were reported, but none of them were related to the vaccine (Logunov et al., 2021).

In an investigation in Argentina, specific antibody response of SARS-CoV-2 was evaluated in a group of naive or previously infected healthcare workers after receiving Sputnik V vaccination, to measure IgG antispike titers and to determine the neutralizing capacity after one and two doses of the vaccine. The majority of naive participants (94%) developed spike-specific IgG antibodies by 3 weeks after receiving the first dose of the vaccine. Unlike the naive group who received two doses of the vaccine, the formerly infected groups receiving a single dose of the vaccine showed higher antibody levels and virus-neutralizing capacity (Rossi et al., 2021). In another study, most of the children (83%) receiving the vaccine developed temporary fever and chills for one or 2 days (Mehraeen et al., 2021). Montalti and associates assessed an AV following immunization (AEFIs) with Sputnik V vaccine. Under study population (*n* = 2,558), with the age range of 18–98 years and the mean age of 66 ± 14 years received one or two doses of the vaccine. Based on their results, first-and second-dose AEFI incidences were 53.3 and 66.8%, respectively. Overall, 76.0% of recipients who received two doses of the vaccine started some AEFIs following either vaccine dose, and 2.1% of subjects aged 60–89 years old experienced severe reactions. In addition, AEFI incidence was 70%, with 53% of the recipients reporting systemic symptoms and 0.8% describing severe reactions. Headache, local and joint pain, and asthenia were the most frequent symptoms (Montalti et al., 2021). Ikegame and colleagues represented the biological results of the ensemble of S mutations in two VOC lineages, i.e., B.1.1.7 (alpha) and B.1.351 (beta) variants. Using replication-competent vesicular stomatitis virus (rcVSV)-CoV2-S, they found out that among 12 serum samples collected from recipients of the Gamaleya Sputnik V Ad26/Ad5 vaccine, only one sample efficiently neutralized rcVSV-CoV2-S:B.1.351 at full serum strength. The same serum also showed effective neutralization of S with B.1.1.7 and moderately decreased activity against S carrying the E484K substitution alone. Their findings demonstrated that vaccines such as Sputnik V are likely to control some emergent SARS-CoV-2 variants (Ikegame et al., 2021). In Venezuela, Claro and co-workers conducted a survey on 86 subjects before and after receiving the Sputnik V vaccine. They determined the IgG antibody response to the nucleocapsid protein and the RBD of the SARS-CoV-2 spike using an ELISA three and 6 weeks after the first and second doses of the Sputnik V vaccine, respectively. Their outcomes revealed that the antibody response in all subjects can be triggered after receiving two doses of the vaccine. Moreover, the second dose of the vaccine did not affect the IgG response for those who were seropositive for SARS-CoV-2 antigens before vaccination (Claro et al., 2021). In one study, the occurrence of early events supposedly attributable to vaccination or immunization (ESAVI) happened in subjects inoculated with the first vaccine dose of Sputnik V. The majority of subjects (71.3%) stated at least one ESAVI; 57% experienced pain at the injection site, 11% reported redness and swelling, 58% stated new or worsened muscle pain, 40% had fever, and 5% suffered from diarrhea. Moreover, serious AV effects were referred by 5%, and one case needed to be hospitalized (Logunov et al., 2021).

Based on the data from phase 3 trials, the result of vaccination was overally effective (Calzetta et al., 2021). Body of evidence has revealed that Sputnik V vaccine has a decreased neutralizing capacity against the beta variant, and all variants with the spike protein carry the E484K substitution. However, Sputnik V vaccine, owing to its characteristics, could be used as a beneficial means of satisfying the need for mass vaccination. Nonetheless, the efficacy and safety of the vaccine are required to be documented (Cazzola et al., 2021).

Some of the most recent studies investigated Sputnik V neutralizing activity against Omicron (B.1.1.529) new variant compared to reference Wuhan D614G (B.1) variant. In a comparison between Sputnik V and BNT162b2 vaccines in people who received two doses of each vaccine, at different time points up to 6 months after administration, Sputnik V showed 8.1-fold decrease in neutralizing antibody (NtAb) that it was 21.4 folds for group of BNT162b2. In addition, 74.2% of Sputnik V and 56.9% of BNT162b2 vaccinated persons had detectable NtAb to SARS-CoV-2 Omicron variant, as a result, Sputnik V can be considered as a booster dose for vaccination with other prime vaccine doses (Lapa et al., 2022). On March 23, 2022 reported Sputnik V had 97% effective against moderate or severe infection development among people living with HIV (PLWH). Overall, 25 trials in 8 countries assess safety and immunization of this vaccine and was approved in 74 countries.

## Viral vector (replicating) vaccine candidates

Viral vector can induce high immunogenicity without an adjuvant and stimulate antigen-specific T cell responses to destroy virus-infected cells. Unlike non-replicating vaccines, these vaccines multiply inside the infected cell and produce more viral antigens. The deletions in nonreplicating viral vector vaccines are not present in replicating viral vectors. Thus, producing S protein in the body gives a better immune response. This type of vaccine has long been used successfully against Newcastle and infectious bursal diseases. There are two replicating viral vector vaccines under phase 3 clinical trials to assess their safety and efficacy. The reporting of efficacy trials and treatment-related SAE have not been published.

### Brilife (IIBR-100) vaccine

The recombinant vesicular tomatitis virus (rVSV)-∆G-spike vaccine named ‘Brilife’ (IIBR-100) is a vaccine in clinical trials developed by the Institute for Biological Research (IIBR) and express the spike protein of the SARS-CoV-2 betacoronavirus on its envelope (Table 4). The rVSV was formerly used as a vaccine platform was designed by John Rose and Michael Whitt for various viral pathogens such as Ebola virus, AIDS (HIV), and Crimean-Congo Hemorrhagic Fever (Suder et al., 2018; Rodriguez et al., 2019). As a vaccine platform, rVSV has various superiorities: is simply propagated and reaches high titers, induces robust cellular and humoral immunity *in vivo*, eliminates the main virulence factor of the VSV, i.e., VSV-G protein, and reduces the virus and its reactogenicity; the Pre-clinical data demonstrated that rVSV-ΔG-SARS-CoV-2-S was a safe vaccine and induced rapidly and strongly neutralized antibodies against SARS-CoV-2 (Yahalom-Ronen et al., 2020; Madar-Balakirski et al., 2021). The immunogenicity, potential efficacy, and safety of the vaccine were evaluated in a randomized, multi-center, controlled phase I/II clinical trial study (NCT04608305). The study is comprised of two phases; phase 1 assesses dose-escalation on 1,040 healthy adults aged 18–55 years. All subjects randomly receive a single injection of IIBR-100 at a low, mid, and high dose or saline or two injections of IIBR-100 at the low dose, or saline, at a 28 days’ interval. Based on data review obtained during phase I, Phase II trials were conducted with larger cohorts as well as elderly age volunteers (18–85) with similar dose treatment of phase I. Also, the booster dose administration (prime-boost) was implemented when immunogenicity responses were inadequate (Chavda et al., 2022).

**Table 4:** Characteristics of COVID-19 replicating viral vectors vaccines.

**Vaccine name**	**Developer**	**Route of administration/ dose**	**Clinical stage**	**Subunit and structure**	**Adjuvant**	**Efficacy**	**Side effects**	**Reference**
*Brilife (IIBR-100)*	NeuroRx, Inc., in collaboration with Cromos, Brilife Georgia, Israel Institute for Biological Research	IM/1	Phase2b/3	rVSV Antigen:, S protein	Without adjuvant	NR (ongoing)	injection-site pain, swelling, redness, headache, feverishness, fatigue, joint and muscle pain, nausea, arthritis, rash, and abnormal sweating	Levin et al., 2021
*DelNS1-2019-nCoV-RBD-OPT1 vaccine*	Beijing Wantai Biological Pharmacy/ Xiamen University	IN/2	Phase III	H1N1 Influenza virus Antigen: non-specified RBD of S protein	Without adjuvant	NR (ongoing)	No serious adverse events were reported.	Chavda et al., 2022

NR: Not Reported. IM: Intramuscular. IN: Intranasal. LNP: lipid nanoparticle.

In the phase 2b/3 trial (NCT04990466), IIBR partnered with the US-based NRx Pharmaceuticals (Cromos, Brilife, Georgia) to complete late-stage clinical studies and mass immunization of the Georgian population in the year 2021. A randomized, multi-center, and observer-blind study was conducted on 550 volunteers in the age group of 18–90 with two intramuscular (IM) injections of the IIBR-100 (prime-boost) or an active comparator at days 0 and 28 days, and followed up was performed to 12 months post last vaccination (Chavda et al., 2022). NRx announced that this vaccine not only does induce a single dose of a vaccine protecting the volunteers from SARS-CoV-2 challenge but also induces adequate levels of neutralizing antibodies against the Omicron. So far, 4 trials in 1 country conducted to assess their safety and efficacy, but this vaccine is not yet approved. This vaccine is ongoing, and data from efficacy trials and treatment-related serious adverse events (SAE) have not been published.

### DelNS1-2019-nCoV-RBD-OPT1 vaccine

This is a two-dose intranasal spray vaccine based on a live attenuated influenza virus-vectored vaccine targeting RBD produced by University of Hong Kong, Xiamen University, and Beijing Wantai Biological Pharmacy. The results of phase I (ChiCTR2000037782) on 60 healthy subjects and phase II (chiCTR2000039715) on 720 healthy subjects clinical indicated that the vaccine is very tolerated and immunogenic in inducing extensive adaptive immune responses, especially at the generation of mucosal IgA, systemic IgG, and T cell responses. Based on promising results obtained during phase I and phase II clinical trials, the phase III trial (ChiCTR2100051391) was conducted in the Philippines involving 40,000 subjects (Chavda et al., 2022). Phase III trial is under investigation, and no data has been reported on therapeutic efficacy, an adjuvant used, and treatment-related SAE. So far, 5 trials in 3 countries, conducted to assess their safety and efficacy.

## Conclusion

Production of effective vaccines based on new platforms like nucleic acid and viral vectors against SARS-CoV-2 infection proved to be a useful option. Herein, we reviewed 19 vaccines passing phase 3 and 4 clinical trial from different countries. SARS-CoV-2 spike protein S is a large trimeric glycoprotein that plays the most essential roles in viral attachment, fusion, and entry and could be considered as a target for the development of antibodies, entry inhibitors, and vaccines. In these vaccines, the main target is the expression of genes that encode the SARS-CoV-2 spike glycoprotein on the host cell surface and then induce humoral and cellular immune responses. The most reported side effects were fever and muscular pain at the injection site. RNA vaccines have an acceptable safety profile because they do not integrate it into host DNA. However, the appropriate temperature to maintain these vaccines with high efficacy is −20°C to −70°C, and providing such an environment is a challenging issue. The long-term immunity resulted by the RNA vaccine remains unknown. There is also some evidence that the immunity derived from these vaccines could decrease, especially against new variants such as delta. Therefore, more studies are needed to evaluate a consensus regarding the different properties of RNA vaccine. Additionally, DNA vaccines advantages are that they can be simply and rapidly produced by synthetic methods or PCR and at a large scale, are safer and more thermostable than other vaccine platforms. On the other hand, one of the most important concern about DNA vaccines is the possibility that the injected DNA will actually integrate into one of the human chromosomes inside the cell. The effect could range from no effect or could potentially lead to cancer through affecting genes controlling cell growth. Overall, the occurrence of severe adverse events in all reviewed vaccines was uncommon or minimal. The efficacy of the vaccines was variable depending on the trial phase and participants’ underlying features. In conclusion, despite all efforts to produce effective vaccines, the appearance of new mutated SARS-CoV-2 strains is a serious challenge. Present vaccine proficiency must be evaluated, and their efficiency have to be reported.

## Author contributions

SK, RG, MS, NS, HG, and MH contributed to the comprehensive research and wrote the manuscript. AS and MS edited the manuscript. All authors contributed to the article and approved the submitted version.

## Conflict of interest

The authors declare that the research was conducted in the absence of any commercial or financial relationships that could be construed as a potential conflict of interest.

## Publisher’s note

All claims expressed in this article are solely those of the authors and do not necessarily represent those of their affiliated organizations, or those of the publisher, the editors and the reviewers. Any product that may be evaluated in this article, or claim that may be made by its manufacturer, is not guaranteed or endorsed by the publisher.

## References

[ref1] Abadi A. H. Mahdavi M. Khaledi A. Esmaeili S.-A. Esmaeili D. Sahebkar A. (2018). Study of serum bactericidal and splenic activity of Total-OMP-CagA combination from Brucella abortus and helicobacter pylori in BALB/c mouse model. Microb. Pathog. 121, 100–105. doi: 10.1016/j.micpath.2018.04.050, PMID: 29709690

[ref2] Ahn J. Y. Lee J. Suh Y. S. Song Y. G. Choi Y.-J. Lee K. H.. (2022). Safety and immunogenicity of two recombinant DNA COVID-19 vaccines containing the coding regions of the spike or spike and nucleocapsid proteins: an interim analysis of two open-label, non-randomised, phase 1 trials in healthy adults. The Lancet Microbe. 3, e173–e183. doi: 10.1016/S2666-5247(21)00358-X, PMID: 35156068 PMC8824525

[ref3] Al Kaabi N. Zhang Y. Xia S. Yang Y. Al Qahtani M. M. Abdulrazzaq N.. (2021). Effect of 2 inactivated SARS-CoV-2 vaccines on symptomatic COVID-19 infection in adults: A randomized clinical trial. JAMA 326, 35–45. doi: 10.1001/jama.2021.8565, PMID: 34037666 PMC8156175

[ref4] Alghamdi A. A. Alkazemi A. Alissa A. Alghamdi I. Alwarafi G. Waggas H. A. (2022). Adverse events following AstraZeneca COVID-19 vaccine in Saudi Arabia: A Cross-sectional study among healthcare and nonhealthcare workers. Intervirology 65, 104–109. doi: 10.1159/000519456, PMID: 34544075 PMC8678246

[ref5] AstraZeneca (2021). First COVID-19 variant vaccine AZD2816 phase II/III trial participants vaccinated. Available at: https://www.astrazeneca.com/media-centre/press-releases/2021/first-covid-19-variant-vaccine-azd2816-phase-ii-iii-trial-participants-vaccinated.html

[ref6] Awadasseid A. Wu Y. Tanaka Y. Zhang W. (2021). Current advances in the development of SARS-CoV-2 vaccines. Int. J. Biol. Sci. 17, 8–19. doi: 10.7150/ijbs.52569, PMID: 33390829 PMC7757035

[ref7] Babamahmoodi F. Saeedi M. Alizadeh-Navaei R. Hedayatizadeh-Omran A. Mousavi S. A. Ovaise G.. (2021). Side effects and immunogenicity following administration of the Sputnik V COVID-19 vaccine in health care workers in Iran. Sci. Rep. 11, 1, 21464–8. doi: 10.1038/s41598-021-00963-7, PMID: 34728696 PMC8563977

[ref8] Baden L. R. El Sahly H. M. Essink B. Kotloff K. Frey S. Novak R.. (2021). Efficacy and safety of the mRNA-1273 SARS-CoV-2 vaccine. N. Engl. J. Med. 384, 403–416. doi: 10.1056/NEJMoa2035389, PMID: 33378609 PMC7787219

[ref9] Bahador A. Esmaeili D. Mansoori N. Mahdavi M. (2013). Protection against Brucellaabortus 544 strain infection in BALB/c mice by subcutaneouse administration of multicomponent vaccine of rCagA conjugated with LPS plus CpG. J Pure. Appl. Microbiol. 7, 1809–1819.

[ref10] Baker N. Dolgin E. (2021). Coronapod: CureVac disappoints in COVID vaccine trial. Nature:5. doi: 10.1038/d41586-021-01694-5 34145414

[ref11] Belete T. M. (2022). The immune response, safety, and efficacy of emergency use authorization-granted COVID-19 vaccines: A review. Open Microbiol. J. 16:1240. doi: 10.2174/18742858-v16-e2201240

[ref12] Borobia A. M. Carcas A. J. Pérez-Olmeda M. Castaño L. Bertran M. J. García-Pérez J.. (2021). Immunogenicity and reactogenicity of BNT162b2 booster in ChAdOx1-S-primed participants (CombiVacS): A multicentre, open-label, randomised, controlled, phase 2 trial. Lancet 398, 121–130. doi: 10.1016/S0140-6736(21)01420-3, PMID: 34181880 PMC8233007

[ref13] Calzetta L. Ritondo B. L. Coppola A. Matera M. G. Di Daniele N. Rogliani P. (2021). Factors influencing the efficacy of COVID-19 vaccines: A quantitative synthesis of phase III trials. Vaccines (Basel). 9 10.3390/vaccines9040341 PMC8065664 33916222

[ref14] Cao Q. Wu S. Xiao C. Chen S. Chi X. Cui X.. (2021). Integrated single-cell analysis revealed immune dynamics during Ad5-nCoV immunization. Cell discovery. 7, 1–17. doi: 10.1038/s41421-021-00300-2 34373443 PMC8352953

[ref15] Capone S. Raggioli A. Gentile M. Battella S. Lahm A. Sommella A.. (2021). Immunogenicity of a new gorilla adenovirus vaccine candidate for COVID-19. Mol. Ther. 29, 2412–2423. doi: 10.1016/j.ymthe.2021.04.022, PMID: 33895322 PMC8062434

[ref16] Cazzola M. Rogliani P. Mazzeo F. Matera M. G. (2021). Controversy surrounding the Sputnik V vaccine. Respir. Med. 187:106569. doi: 10.1016/j.rmed.2021.106569, PMID: 34399368 PMC8352655

[ref17] CDC. (n.d.). Available at: Accines\covid-19\hcp\viral-Vector-Vaccine-Basics.Html-Hwcgc-nvdvhCArhAFFcgF.

[ref18] Chang C. C. Crane M. Zhou J. Mina M. Post J. J. Cameron B. A.. (2013). HIV and co-infections. Immunol. Rev. 254, 114–142. doi: 10.1111/imr.12063, PMID: 23772618 PMC3697435

[ref19] Chary M. A. Barbuto A. F. Izadmehr S. Hayes B. D. Burns M. M. (2020). COVID-19: therapeutics and their toxicities. J. Med. Toxicol. 16, 284–294. doi: 10.1007/s13181-020-00777-5, PMID: 32356252 PMC7192319

[ref20] Chaudhary N. Weissman D. Whitehead K. A. (2021). mRNA vaccines for infectious diseases: principles, delivery and clinical translation. Nat. Rev. Drug Discov. 20, 817–838. doi: 10.1038/s41573-021-00283-5 34433919 PMC8386155

[ref21] Chauhan N. Soni S. Gupta A. Aslam M. Jain U. (2021). Interpretative immune targets and contemporary position for vaccine development against SARS-CoV-2: A systematic review. J. Med. Virol. 93, 1967–1982. doi: 10.1002/jmv.26709, PMID: 33270225 PMC7753271

[ref22] Chavda V. P. Bezbaruah R. Athalye M. Parikh P. K. Chhipa A. S. Patel S.. (2022). Replicating viral vector-based vaccines for COVID-19: potential avenue in vaccination arena. Viruses 14, 759. doi: 10.3390/v14040759, PMID: 35458489 PMC9025561

[ref23] Chavda V. P. Pandya R. Apostolopoulos V. (2021). DNA vaccines for SARS-CoV-2: toward third-generation vaccination era. Expert Rev. Vaccines 20, 1549–1560. doi: 10.1080/14760584.2021.1987223, PMID: 34582298 PMC8567274

[ref24] Chemaitelly H. Yassine H. M. Benslimane F. M. Al Khatib H. A. Tang P. Hasan M. R.. (2021). mRNA-1273 COVID-19 vaccine effectiveness against the B.1.1.7 and B.1.351 variants and severe COVID-19 disease in Qatar. Nat. Med. 27, 1614–1621. doi: 10.1038/s41591-021-01446-y, PMID: 34244681

[ref25] Clark A. Jit M. Warren-Gash C. Guthrie B. Wang H. H. Mercer S. W.. (2020). Global, regional, and national estimates of the population at increased risk of severe COVID-19 due to underlying health conditions in 2020: A modelling study. Lancet Glob. Health 8, e1003–e1017. doi: 10.1016/S2214-109X(20)30264-3, PMID: 32553130 PMC7295519

[ref26] Claro F. Silva D. Rodriguez M. Rangel R. de Waard J. H. (2021). IgG antibody response to the Sputnik V vaccine: previous SARS-CoV-2 seropositive individuals might need just one vaccine dose. Int. J. Infect. Dis. 111, 261–266. doi: 10.1016/j.ijid.2021.07.070, PMID: 34343704 PMC8325383

[ref27] Clinical Trials Arena (2021). Arcturus to initiate trial of Covid-19 vaccine in Vietnam. Available at: https://www.clinicaltrialsarena.com/news/arcturus-trial-covid-vaccine/

[ref28] ClinicalTrials.gov. (2021a). A phase III clinical study of a SARS-CoV-2 messenger ribonucleic acid (mRNA) vaccine candidate Against COVID-19 in population aged 18 years and Above. Available at: https://clinicaltrials.gov/ct2/show/NCT04847102

[ref29] ClinicalTrials.gov. (2021b). Study of GRAd-COV2 for the prevention of COVID-19 in adults (COVITAR). Available at: https://clinicaltrials.gov/ct2/show/study/NCT04791423

[ref30] ClinicalTrials.gov. (2022a). A study to evaluate the immunogenicity and safety of mRNA vaccine boosters for SARS-CoV-2 (COVID-19) variants. Available at: https://clinicaltrials.gov/ct2/show/study/NCT04927065

[ref31] Clinicaltrials.gov. (2022b). Safety and immunogenicity study of a SARS-CoV-2 (COVID-19) variant vaccine (mRNA-1273.351) in Naïve and previously vaccinated adults. Available at: https://clinicaltrials.gov/ct2/show/study/NCT04785144

[ref32] ClinicalTrials.gov. (2022c). The ARCT-154 self-amplifying RNA vaccine efficacy study (ARCT-154-01) (ARCT-154-01). Available at: https://clinicaltrials.gov/ct2/show/NCT05012943

[ref33] Corbett K. S. Flynn B. Foulds K. E. Francica J. R. Boyoglu-Barnum S. Werner A. P.. (2020). Evaluation of the mRNA-1273 vaccine against SARS-CoV-2 in nonhuman primates. N. Engl. J. Med. 383, 1544–1555. doi: 10.1056/NEJMoa2024671, PMID: 32722908 PMC7449230

[ref34] Corbett K. S. Nason M. Flach B. Gagne M. O'Connell S. Johnston T.. (2021). Immune correlates of protection by mRNA-1273 immunization against SARS-CoV-2 infection in nonhuman primates. bioRxiv. 10.1126/science.abj0299 PMC8449013 34529476

[ref35] Cromer D. Reynaldi A. Steain M. Triccas J. A. Davenport M. P. Khoury D. S. (2021). Relating in vitro neutralisation level and protection in the CVnCoV (CUREVAC) trial. medRxiv. 10.1093/cid/ciac075 PMC9383389 35100611

[ref36] DeJesus E. Herrera G. Teofilo E. Gerstoft J. Buendia C. B. Brand J. D.. (2004). Abacavir versus zidovudine combined with lamivudine and efavirenz, for the treatment of antiretroviral-naive HIV-infected adults. Clin. Infect. Dis. 39, 1038–1046. doi: 10.1086/424009, PMID: 15472858

[ref37] Dey A. Rajanathan C. Chandra H. Pericherla H. P. Kumar S. Choonia H. S.. (2021). Immunogenic potential of DNA vaccine candidate, ZyCoV-D against SARS-CoV-2 in animal models. bioRxiv. 10.1016/j.vaccine.2021.05.098 PMC8166516 34120764

[ref38] Folegatti P. M. Ewer K. J. Aley P. K. Angus B. Becker S. Belij-Rammerstorfer S.. (2020). Safety and immunogenicity of the ChAdOx1 nCoV-19 vaccine against SARS-CoV-2: a preliminary report of a phase 1/2, single-blind, randomised controlled trial. Lancet 396, 467–478. doi: 10.1016/S0140-6736(20)31604-4, PMID: 32702298 PMC7445431

[ref39] Frenck R. W. Jr. Klein N. P. Kitchin N. Gurtman A. Absalon J. Lockhart S.. (2021). Safety, immunogenicity, and efficacy of the BNT162b2 Covid-19 vaccine in adolescents. N. Engl. J. Med. 385, 239–250. doi: 10.1056/NEJMoa2107456, PMID: 34043894 PMC8174030

[ref40] Funk C. D. Laferrière C. Ardakani A. (2020). A snapshot of the global race for vaccines targeting SARS-CoV-2 and the COVID-19 pandemic. Front. Pharmacol. 11:937. doi: 10.3389/fphar.2020.00937, PMID: 32636754 PMC7317023

[ref41] Genetic Engineering & Biotechnology News. (2022). Available at: https://www.genengnews.com/covid-19-candidates/moderna/

[ref42] Granados-Riveron J. T. Aquino-Jarquin G. (2021). Engineering of the current nucleoside-modified mRNA-LNP vaccines against SARS-CoV-2. Biomed. Pharmacother. 142, 111953. doi: 10.1016/j.biopha.2021.111953 34343897 PMC8299225

[ref43] Guzmán-Martínez O. Guardado K. de Guevara E. L. Navarro S. Hernández C. Zenteno-Cuevas R.. (2021). IgG antibodies generation and side effects caused by Ad5-nCoV vaccine (CanSino biologics) and BNT162b2 vaccine (Pfizer/BioNTech) among Mexican population. Vaccine 9, 999. doi: 10.3390/vaccines9090999, PMID: 34579236 PMC8473118

[ref44] Halperin S. A. Ye L. MacKinnon-Cameron D. Smith B. Cahn P. E. Ruiz-Palacios G. M.. (2022). Final efficacy analysis, interim safety analysis, and immunogenicity of a single dose of recombinant novel coronavirus vaccine (adenovirus type 5 vector) in adults 18 years and older: an international, multicentre, randomised, double-blinded, placebo-controlled phase 3 trial. Lancet 399, 237–248. doi: 10.1016/S0140-6736(21)02753-7, PMID: 34953526 PMC8700283

[ref45] Heidary M. Kaviar V. H. Shirani M. Ghanavati R. Motahar M. Sholeh M.. (2022). A comprehensive review of the protein subunit vaccines Against COVID-19. Front. Microbiol. 13:306. doi: 10.3389/fmicb.2022.927306 PMC9329957 35910658

[ref46] Hernández-Bello J. Morales-Núñez J. J. Machado-Sulbarán A. C. Díaz-Pérez S. A. Torres-Hernández P. C. Balcázar-Félix P.. (2021). Neutralizing antibodies against SARS-CoV-2, anti-Ad5 antibodies, and Reactogenicity in response to Ad5-nCoV (CanSino biologics) vaccine in individuals with and without prior SARS-CoV-2. Vaccine 9:1047. doi: 10.3390/vaccines9091047, PMID: 34579284 PMC8472849

[ref47] Hoffmann D. Corleis B. Rauch S. Roth N. Mühe J. Halwe N. J.. (2021). CVnCoV and CV2CoV protect human ACE2 transgenic mice from ancestral B BavPat1 and emerging B.1.351 SARS-CoV-2. Nature. Communications 12, 4048. doi: 10.1038/s41467-021-24339-7 PMC8245475 34193869

[ref48] Ikegame S. Siddiquey M. N. A. Hung C. T. Haas G. Brambilla L. Oguntuyo K. Y.. (2021). Neutralizing activity of Sputnik V vaccine sera against SARS-CoV-2 variants. Res Sq. 10.1038/s41467-021-24909-9 PMC8313705 34312390

[ref49] Investors. (2020). Available at: https://investors.modernatx.com/news-releases/news-release-details/modernas-covid-19-vaccine-candidate-meets-its-primary-efficacy (accessed on 27 November 2020). MMsC-VCMIPEEitFIAotPCSAo.

[ref50] Jackson L. A. Anderson E. J. Rouphael N. G. Roberts P. C. Makhene M. Coler R. N.. (2020). An mRNA vaccine against SARS-CoV-2—preliminary report. N. Engl. J. Med. 383, 1920–1931. doi: 10.1056/NEJMoa2022483, PMID: 32663912 PMC7377258

[ref51] Kaur S. P. Gupta V. (2020). COVID-19 Vaccine: A comprehensive status report. Virus Res. 198114. 10.1016/j.virusres.2020.198114 PMC7423510 32800805

[ref52] Khavari A. Orafa Z. Hashemi M. Habibzadeh N. Bolhassani A. (2016). Different physical delivery systems: An important approach for delivery of biological molecules in vivo. Arch. Adv.n Biosci. 7, 48–63.

[ref53] Khobragade A. Bhate S. Ramaiah V. Deshpande S. Giri K. Phophle H.. (2022). Efficacy, safety, and immunogenicity of the DNA SARS-CoV-2 vaccine (ZyCoV-D): the interim efficacy results of a phase 3, randomised, double-blind, placebo-controlled study in India. Lancet 399, 1313–1321. doi: 10.1016/S0140-6736(22)00151-9, PMID: 35367003 PMC8970574

[ref54] Khoshnood S. Arshadi M. Akrami S. Koupaei M. Ghahramanpour H. Shariati A.. (2022). An overview on inactivated and live-attenuated SARS-CoV-2 vaccines. J. Clin. Lab. Anal. 36:e24418. doi: 10.1002/jcla.24418, PMID: 35421266 PMC9102488

[ref55] Khoury D. S. Cromer D. Reynaldi A. Schlub T. E. Wheatley A. K. Juno J. A.. (2021). Neutralizing antibody levels are highly predictive of immune protection from symptomatic SARS-CoV-2 infection. Nat. Med. 27, 1205–1211. doi: 10.1038/s41591-021-01377-8 34002089

[ref56] Kim J. H. Jacob J. (2009). DNA vaccines against influenza viruses. Vacc. Pand. Influ., 197–210. doi: 10.1007/978-3-540-92165-3_10 19768407

[ref57] Kontou E. Ranellou K. Zoulas D. Bletsa A. Rompola E. Piperaki E.-T.. (2021). Antibody response following a two-dose mRNA vaccination regimen, in health Care Workers of a Tertiary Hospital in Athens, Greece. J. Person. Med. 11, 576. doi: 10.3390/jpm11060576, PMID: 34205301 PMC8234272

[ref58] Krammer F. (2020). SARS-CoV-2 vaccines in development. Nature 586, 516–527. doi: 10.1038/s41586-020-2798-3 32967006

[ref59] Kremsner P. G. Ahuad Guerrero R. A. Arana-Arri E. Aroca Martinez G. J. Bonten M. Chandler R.. (2022). Efficacy and safety of the CVnCoV SARS-CoV-2 mRNA vaccine candidate in ten countries in Europe and Latin America (HERALD): a randomised, observer-blinded, placebo-controlled, phase 2b/3 trial. Lancet Infect. Dis. 22, 329–340. doi: 10.1016/S1473-3099(21)00677-0, PMID: 34826381 PMC8610426

[ref60] Kremsner P. G. Mann P. Kroidl A. Leroux-Roels I. Schindler C. Gabor J. J.. (2021). Safety and immunogenicity of an mRNA-lipid nanoparticle vaccine candidate against SARS-CoV-2: A phase 1 randomized clinical trial. Wien. Klin. Wochenschr., 1–11. 10.1007/s00508-021-01922-y PMC8354521 34378087

[ref61] Lanini S. Capone S. Antinori A. Milleri S. Nicastri E. Camerini R.. (2021b). GRAd-COV2, a gorilla adenovirus-based candidate vaccine against COVID-19, is safe and immunogenic in younger and older adults. Sci. Transl. Med. 14:eabj1996. doi: 10.1126/scitranslmed.abj1996 34698501

[ref62] Lanini S. Capone S. Antinori A. Milleri S. Nicastri E. Camerini R.. (2021a). GRAd-COV2, a gorilla adenovirus based candidate vaccine against COVID-19, is safe and immunogenic in young and older adults. medRxiv10.1126/scitranslmed.abj1996 34698501

[ref63] Lapa D. Grousova D. Matusali G. Meschi S. Colavita F. Bettini A.. (2022). Retention of neutralizing response against SARS-CoV-2 omicron variant in Sputnik V vaccinated individuals. medRxiv. 10.3390/vaccines10050817 PMC9144866 35632574

[ref64] Lefebvre M. Vignier N. Pitard B. Botelho-Nevers E. Cohen R. Epaulard O. (2021). Covid-19 vaccines: frequently asked questions and updated answers. Infect. Dis. Now. 51, 319–333. doi: 10.1016/j.idnow.2021.02.007, PMID: 33681861 PMC7910656

[ref65] Levin Y. Balakirski N. M. Caraco Y. Ben-Ami E. Atsmon J. Marcus H. (2021). Ethics and execution of developing a 2nd wave COVID vaccine–our interim phase I/II VSV-SARS-CoV2 vaccine experience. Vaccine 39, 2821–2823. doi: 10.1016/j.vaccine.2021.04.017, PMID: 33896663 PMC8043614

[ref66] Liu M. A. (2019). A comparison of plasmid DNA and mRNA as vaccine technologies. Vaccine 7:37. doi: 10.3390/vaccines7020037, PMID: 31022829 PMC6631684

[ref67] Logunov D. Y. Dolzhikova I. V. Shcheblyakov D. V. Tukhvatulin A. I. Zubkova O. V. Dzharullaeva A. S.. (2021). Safety and efficacy of an rAd26 and rAd5 vector-based heterologous prime-boost COVID-19 vaccine: An interim analysis of a randomised controlled phase 3 trial in Russia. Lancet 397, 671–681. doi: 10.1016/S0140-6736(21)00234-8, PMID: 33545094 PMC7852454

[ref68] Lustig Y. Zuckerman N. Nemet I. Atari N. Kliker L. Regev-Yochay G.. (2021). Neutralising capacity against Delta (B. 1.617. 2) and other variants of concern following Comirnaty (BNT162b2, BioNTech/Pfizer) vaccination in health care workers, Israel. Eur. Secur. 26:2100557. 10.2807/1560-7917.ES.2021.26.26.2100557 PMC8326656 34212838

[ref69] Madar-Balakirski N. Rosner A. Melamed S. Politi B. Steiner M. Tamir H.. (2021). Nonclinical safety and immunogenicity of an rVSV-ΔG-SARS-CoV-2-S vaccine in mice, hamsters, rabbits and pigs. bioRxiv. 10.1007/s00204-021-03214-w PMC8760087 35032184

[ref70] Madhi S. A. Baillie V. Cutland C. L. Voysey M. Koen A. L. Fairlie L.. (2021). Efficacy of the ChAdOx1 nCoV-19 Covid-19 vaccine against the B. 1.351 variant. N. Engl. J. Med. 384, 1885–1898. doi: 10.1056/NEJMoa2102214, PMID: 33725432 PMC7993410

[ref71] Mehraeen E. SeyedAlinaghi S. Karimi A. (2021). Can children of the Sputnik V vaccine recipients become symptomatic? Hum. Vaccin. Immunother. 17, 3500–3501. doi: 10.1080/21645515.2021.1933689, PMID: 34241575 PMC8290368

[ref72] Mercado N. B. Zahn R. Wegmann F. Loos C. Chandrashekar A. Yu J.. (2020). Single-shot Ad26 vaccine protects against SARS-CoV-2 in rhesus macaques. Nature 586, 583–588. doi: 10.1038/s41586-020-2607-z, PMID: 32731257 PMC7581548

[ref73] Montalti M. Soldà G. Di Valerio Z. Salussolia A. Lenzi J. Forcellini M.. (2021). ROCCA observational study: early results on safety of Sputnik V vaccine (gam-COVID-Vac) in the Republic of San Marino using active surveillance. EClinicalMedicine. 38:101027. doi: 10.1016/j.eclinm.2021.101027, PMID: 34505029 PMC8413252

[ref74] Motamedi H. Ari M. M. Dashtbin S. Fathollahi M. Hossainpour H. Alvandi A.. (2021). An update review of globally reported SARS-CoV-2 vaccines in preclinical and clinical stages. Int. Immunopharmacol. 96:107763. doi: 10.1016/j.intimp.2021.107763, PMID: 34162141 PMC8101866

[ref75] Naygovzina N. B. Khabriev R. U. Krasheninnikov A. E. Matveev A. V. (2021). The organizational aspects of security support of participants of clinical testing of vaccine "gam-COVID-Vac". Probl Sotsialnoi Gig Zdravookhranenniiai Istor Med. 29, 5–13. doi: 10.32687/0869-866X-2021-29-1-5-13, PMID: 33591649

[ref76] Ndwandwe D. Wiysonge C. S. (2021). COVID-19 vaccines. Curr. Opin. Immunol. 71, 111–116. doi: 10.1016/j.coi.2021.07.003, PMID: 34330017 PMC8272971

[ref77] Otto S. P. Day T. Arino J. Colijn C. Dushoff J. Li M.. (2021). The origins and potential future of SARS-CoV-2 variants of concern in the evolving COVID-19 pandemic. Curr. Biol. 31, R918–R929. doi: 10.1016/j.cub.2021.06.049, PMID: 34314723 PMC8220957

[ref78] Park J. W. Lagniton P. N. P. Liu Y. Xu R. H. (2021). mRNA vaccines for COVID-19: what, why and how. Int. J. Biol. Sci. 17, 1446–1460. doi: 10.7150/ijbs.59233, PMID: 33907508 PMC8071766

[ref79] Pfizer (2020). Pfizer and BioNTech announce data from preclinical studies of mRNA-based vaccine candidate Against COVID-19. Available at: https://www.pfizer.com/news/press-release/press-release-detail/pfizer-and-biontech-announce-data-preclinical-studies-mrna

[ref80] Pharmaceutical Technology (2021). Available at: https://www.pharmaceutical-technology.com/news/moderna-covid-19-variant-vaccines-study/

[ref81] Polack F. P. Thomas S. J. Kitchin N. Absalon J. Gurtman A. Lockhart S.. (2020). Safety and efficacy of the BNT162b2 mRNA Covid-19 vaccine. N. Engl. J. Med. 383, 2603–2615. doi: 10.1056/NEJMoa2034577, PMID: 33301246 PMC7745181

[ref82] Poland G. A. Ovsyannikova I. G. Kennedy R. B. (2020). SARS-CoV-2 immunity: review and applications to phase 3 vaccine candidates. Lancet 396, 1595–1606. doi: 10.1016/S0140-6736(20)32137-1, PMID: 33065034 PMC7553736

[ref83] Pormohammad A. Zarei M. Ghorbani S. Mohammadi M. Razizadeh M. H. Turner D. L.. (2021). Efficacy and safety of COVID-19 vaccines: a systematic review and meta-analysis of randomized clinical trials. Vaccine 9, 467. doi: 10.3390/vaccines9050467, PMID: 34066475 PMC8148145

[ref84] Ramasamy M. N. Minassian A. M. Ewer K. J. Flaxman A. L. Folegatti P. M. Owens D. R.. (2020). Safety and immunogenicity of ChAdOx1 nCoV-19 vaccine administered in a prime-boost regimen in young and old adults (COV002): a single-blind, randomised, controlled, phase 2/3 trial. Lancet 396, 1979–1993. doi: 10.1016/S0140-6736(20)32466-1, PMID: 33220855 PMC7674972

[ref85] Rauch S. Gooch K. E. Hall Y. Salguero F. J. Dennis M. J. Gleeson F. V.. (2020). mRNA vaccine CVnCoV protects non-human primates from SARS-CoV-2 challenge infection. Biorxiv.

[ref86] Rauch S. Roth N. Schwendt K. Fotin-Mleczek M. Mueller S. O. Petsch B. (2021). mRNA-based SARS-CoV-2 vaccine candidate CVnCoV induces high levels of virus-neutralising antibodies and mediates protection in rodents. NPJ Vaccines. 6, 57. doi: 10.1038/s41541-021-00311-w, PMID: 33863911 PMC8052455

[ref87] Rodriguez S. E. Cross R. W. Fenton K. A. Bente D. A. Mire C. E. Geisbert T. W. (2019). Vesicular stomatitis virus-based vaccine protects mice against Crimean-Congo hemorrhagic fever. Sci. Rep. 9, 1–13. 31123310 10.1038/s41598-019-44210-6 PMC6533279

[ref88] Romero-Ibarguengoitia M. E. Gonzalez-Cantu A. Hernandez-Ruiz Y. G. Armendariz-Vaquez A. G. Rivera-Salinas D. Montelongo-Cruz L. P.. (2021). Effect of heterologous vaccination regimen with Ad5-nCoV CanSinoBio and BNT162b2 Pfizer in SARS-CoV-2 IgG antibodies titers. medRxiv. 10.3390/vaccines10030392 PMC8948699 35335024

[ref89] Rossi A. H. Ojeda D. S. Varese A. Sanchez L. Gonzalez Lopez Ledesma M. M. Mazzitelli I.. (2021). Sputnik V vaccine elicits seroconversion and neutralizing capacity to SARS-CoV-2 after a single dose. Cell Rep Med. 2, 100359. doi: 10.1016/j.xcrm.2021.100359, PMID: 34308389 PMC8266543

[ref90] Rzymski P. Zeyland J. Poniedziałek B. Małecka I. Wysocki J. (2021). The perception and attitudes toward COVID-19 vaccines: A Cross-sectional study in Poland. Vaccines (Basel). 9:382. doi: 10.3390/vaccines9040382 33919672 PMC8069794

[ref91] Sadoff J. Gray G. Vandebosch A. Cárdenas V. Shukarev G. Grinsztejn B.. (2021). Safety and efficacy of single-dose Ad26. COV2. S vaccine against Covid-19. N. Engl. J. Med. 384, 2187–2201. doi: 10.1056/NEJMoa2101544, PMID: 33882225 PMC8220996

[ref92] Sadoff J. Gray G. Vandebosch A. Cárdenas V. Shukarev G. Grinsztejn B.. (2022). Final analysis of efficacy and safety of single-dose Ad26. COV2. S. N. Engl. J. Med. 386, 847–860. doi: 10.1056/NEJMoa2117608, PMID: 35139271 PMC8849184

[ref93] Sadoff J. Le Gars M. Shukarev G. Heerwegh D. Truyers C. de Groot A. M.. (2021). Interim results of a phase 1–2a trial of Ad26. COV2. S Covid-19 vaccine. N. Engl. J. Med. 384, 1824–1835. doi: 10.1056/NEJMoa2034201, PMID: 33440088 PMC7821985

[ref94] Sahin U. Muik A. Vogler I. Derhovanessian E. Kranz L. M. Vormehr M.. (2021). BNT162b2 vaccine induces neutralizing antibodies and poly-specific T cells in humans. Nature 595, 572–577. doi: 10.1038/s41586-021-03653-6 34044428

[ref95] Sanicas M. Sanicas M. Diop D. Montomoli E. (2020). A review of COVID-19 vaccines in development: 6 months into the pandemic. Pan Afr. Med. J. 37, 124. 33425157 10.11604/pamj.2020.37.124.24973 PMC7755367

[ref96] Sartorius A. G. (2022). Recombinant Protein Vaccine Process. Available at: https://www.sartorius.com/en/applications/biopharmaceutical-manufacturing/vaccines/vaccine-development/recombinant-subunit-vaccines

[ref97] Silveira M. M. Moreira G. Mendonça M. (2021). DNA vaccines against COVID-19: perspectives and challenges. Life Sci. 267:118919. doi: 10.1016/j.lfs.2020.118919, PMID: 33352173 PMC7749647

[ref98] Smith T. R. Patel A. Ramos S. Elwood D. Zhu X. Yan J.. (2020). Immunogenicity of a DNA vaccine candidate for COVID-19. Nat. Commun. 11, 1–13. doi: 10.1038/s41467-020-16505-0, PMID: 32433465 PMC7239918

[ref99] Spencer A. J. Morris S. Ulaszewska M. Powers C. Kailath R. Bissett C.. (2021). The ChAdOx1 vectored vaccine, AZD2816, induces strong immunogenicity against SARS-CoV-2 B.1.351 and other variants of concern in preclinical studies. bioRxiv. 10.1016/j.ebiom.2022.103902 PMC8881183 35228013

[ref100] Strizova Z. Smetanova J. Bartunkova J. Milota T. (2021). Principles and challenges in anti-COVID-19 vaccine development. Int. Arch. Allergy Immunol. 182, 339–349. doi: 10.1159/000514225 33524979 PMC7900461

[ref101] Suder E. Furuyama W. Feldmann H. Marzi A. de Wit E. (2018). The vesicular stomatitis virus-based Ebola virus vaccine: From concept to clinical trials. Hum. Vaccin. Immunother. 14, 2107–2113. doi: 10.1080/21645515.2018.1473698, PMID: 29757706 PMC6183239

[ref102] Sulemankhil I. Abdelrahman M. Negi S. I. (2021). Temporal association between the COVID-19 Ad26. COV2. S vaccine and acute myocarditis: a case report and literature review. Cardiovasc. Revasc. Med. 38, 117–123. doi: 10.1016/j.carrev.2021.08.012 34420869 PMC8364889

[ref103] Tebas P. Yang S. Boyer J. D. Reuschel E. L. Patel A. Christensen-Quick A.. (2021). Safety and immunogenicity of INO-4800 DNA vaccine against SARS-CoV-2: A preliminary report of an open-label, phase 1 clinical trial. EClinicalMedicine. 31:100689. doi: 10.1016/j.eclinm.2020.100689, PMID: 33392485 PMC7759123

[ref104] Tian J.-H. Patel N. Haupt R. Zhou H. Weston S. Hammond H.. (2021). SARS-CoV-2 spike glycoprotein vaccine candidate NVX-CoV2373 immunogenicity in baboons and protection in mice. Nat. Commun. 12, 1–14. 33446655 10.1038/s41467-020-20653-8 PMC7809486

[ref105] Tobaiqy M. MacLure K. Elkout H. Stewart D. (2021). Thrombotic adverse events reported for Moderna, Pfizer and Oxford-AstraZeneca COVID-19 vaccines: comparison of occurrence and clinical outcomes in the EudraVigilance database. Vaccine 9, 1326. doi: 10.3390/vaccines9111326, PMID: 34835256 PMC8624459

[ref106] Tumban E. (2021). Lead SARS-CoV-2 candidate vaccines: expectations from phase III trials and recommendations post-vaccine approval. Viruses 13:54. doi: 10.3390/v13010054 PMC7824305 33396343

[ref107] Ura T. Okuda K. Shimada M. (2014). Developments in viral vector-based vaccines. Vaccine 2, 624–641. doi: 10.3390/vaccines2030624, PMID: 26344749 PMC4494222

[ref108] van Doremalen N. Schulz J. E. Adney D. R. Saturday T. A. Fischer R. J. Yinda C. K.. (2022). Efficacy of ChAdOx1 vaccines against SARS-CoV-2 variants of concern Beta, Delta and omicron in the Syrian hamster model. Res. Sq. 3:rs-1343927.

[ref109] Vietnam News Agency. ARCT-154 vaccine safe for healthy volunteers: initial results. Available at: https://ncov.vnanet.vn/en/news/arct-154-vaccine-safe-for-healthy-volunteers-initial-results/59d2edf7-4989-4c32-878d-5863966aec7e

[ref110] Voysey M. Clemens S. A. C. Madhi S. A. Weckx L. Y. Folegatti P. M. Aley P. K.. (2021). Single-dose administration and the influence of the timing of the booster dose on immunogenicity and efficacy of ChAdOx1 nCoV-19 (AZD1222) vaccine: A pooled analysis of four randomised trials. Lancet 397, 881–891. doi: 10.1016/S0140-6736(21)00432-3, PMID: 33617777 PMC7894131

[ref111] WHO. (2022). Weekly epidemiological update on COVID-19. Available at: https://www.who.int/publications/m/item/weekly-epidemiological-update-on-covid-19---13-july-2022

[ref112] Wu K. Choi A. Koch M. Elbashir S. Ma L. Lee D.. (2021a). Variant SARS-CoV-2 mRNA vaccines confer broad neutralization as primary or booster series in mice. bioRxiv. 10.1016/j.vaccine.2021.11.001 PMC8572694 34815117

[ref113] Wu K. Choi A. Koch M. Ma L. Hill A. Nunna N.. (2021b). Preliminary analysis of safety and immunogenicity of a SARS-CoV-2 variant vaccine booster. Fortschr. Med. 10.1038/s41591-021-01527-y PMC8604720 34526698

[ref114] Wu S. Huang J. Zhang Z. Wu J. Zhang J. Hu H.. (2021c). Safety, tolerability, and immunogenicity of an aerosolised adenovirus type-5 vector-based COVID-19 vaccine (Ad5-nCoV) in adults: preliminary report of an open-label and randomised phase 1 clinical trial. Lancet Infect. Dis. 21, 1654–1664. doi: 10.1016/S1473-3099(21)00396-0, PMID: 34324836 PMC8313090

[ref115] Wu S. Zhong G. Zhang J. Shuai L. Zhang Z. Wen Z.. (2020). A single dose of an adenovirus-vectored vaccine provides protection against SARS-CoV-2 challenge. Nat. Commun. 11, 1–7. 32796842 10.1038/s41467-020-17972-1 PMC7427994

[ref116] Yahalom-Ronen Y. Tamir H. Melamed S. Politi B. Shifman O. Achdout H.. (2020). A single dose of Recombinant VSV-{Triangleup} G-Spike Vaccine Provides Protection against SARS-CoV-2 Challenge. Nat. Commun. 11:6402. doi: 10.1038/s41467-020-20228-7 33328475 PMC7745033

[ref117] Ye T. Zhong Z. García-Sastre A. Schotsaert M. De Geest B. G. (2020). Current status of COVID-19 (pre) clinical vaccine development. Angew. Chem. Int. Ed. 59, 18885–18897. doi: 10.1002/anie.202008319, PMID: 32663348 PMC7405471

[ref118] Ying B. Whitener B. VanBlargan L. A. Hassan A. O. Shrihari S. Liang C.-Y.. (2021). Protective activity of mRNA vaccines against ancestral and variant SARS-CoV-2 strains. bioRxiv. 10.1126/scitranslmed.abm3302 PMC8817234 34846168

[ref119] Zeng C. Zhang C. Walker P. G. Dong Y. (2020). Formulation and delivery technologies for mRNA vaccines. Curr. Top. Microbiol. Immunol.:217. doi: 10.1007/82_2020_217 PMC8195316 32483657

[ref120] Zhang N.-N. Li X.-F. Deng Y.-Q. Zhao H. Huang Y.-J. Yang G.. (2020). A Thermostable mRNA vaccine against COVID-19. Cell 182, 1271–83.e16. doi: 10.1016/j.cell.2020.07.024, PMID: 32795413 PMC7377714

[ref121] Zhu F.-C. Guan X.-H. Li Y.-H. Huang J.-Y. Jiang T. Hou L.-H.. (2020). Immunogenicity and safety of a recombinant adenovirus type-5-vectored COVID-19 vaccine in healthy adults aged 18 years or older: A randomised, double-blind, placebo-controlled, phase 2 trial. Lancet 396, 479–488. doi: 10.1016/S0140-6736(20)31605-6, PMID: 32702299 PMC7836858

[ref122] Zhu H. Wei L. Niu P. (2020). The novel coronavirus outbreak in Wuhan, China. Global Health Res. Policy 5:6. doi: 10.1186/s41256-020-00135-6, PMID: 32226823 PMC7050114

